# 8u, a pro-apoptosis/cell cycle arrest compound, suppresses invasion and metastasis through HSP90α downregulating and PI3K/Akt inactivation in hepatocellular carcinoma cells

**DOI:** 10.1038/s41598-017-18701-3

**Published:** 2018-01-10

**Authors:** Ning Wang, Shaopeng Chen, Bin Zhang, Shangfu Li, Feng Jin, Dan Gao, Hongxia Liu, Yuyang Jiang

**Affiliations:** 1 Department of Chemistry, Tsinghua University, Beijing, 100084 China; 2 State Key Laboratory Breeding Base-Shenzhen Key Laboratory of Chemical Biology, Graduate School at Shenzhen, Tsinghua University, Shenzhen, 518055 China; 3 Li Dak Sum Yip Yio Chin Kenneth Li Marine Biopharmaceutical Research Center, Ningbo University, Ningbo, 315211 China; 4 Neptunus Pharmaceutical Technology Center, Shenzhen, 518057 China; 5 School of Medicine, Tsinghua University, Beijing, 100084 China

## Abstract

8u, an acridine derivative, has been proved effective anti-hepatocarcinoma effect, while the underlying mechanism remains unclear. Here, metabolomics and proteomics approaches were applied to study its anti-cancer mechanism and explore its effect on HepG2 cells’ invasion and metastasis abilities. The results showed that 8u significantly suppressed HepG2 cells migration and enhanced cell-to-cell junctions. The inhibition effect of 8u on invasion and metastasis disappeared after HSP90α gene silencing, and was reversed after HSP90α overexpression. The biological experimental results indicated that 8u also blocked PI3K/Akt pathway, thereby reducing fatty acid synthase (FASN) protein expression and disordering intracellular lipid metabolism to inhibit cell invasion and metastasis. In addition, HSP90α protein and PI3K/Akt pathway could co-adjust to each other. These findings demonstrated that 8u could efficiently suppress the invasion and metastasis of HepG2 cells by decreasing the expression of HSP90α protein and inhibiting the PI3K/Akt signaling pathway, which could be used as a potential candidate for the treatment of HCC.

## Introduction

Hepatocellular carcinoma (HCC) is the sixth most common potentially lethal human malignancy in the world, which is characterized by high morbidity and mortality^1^. So far, HCC is still an incurable disease, because it has strong abilities of invasion and metastasis^2^. Currently, therapies for HCC include chemical therapy, surgical resection, partial ablation therapy, and liver transplantation^3–6^. However, recurrence and metastasis after surgery, as well as drug resistance are major barriers to successful therapy, thus leading to a poor outcome in HCC patients^7^. Sorafenib, a multikinase inhibitor, approved by FDA for the treatment of advanced HCC. However, it has only slight survival advantage compared with its major side effects^8^. At present, anti-hepatocarcinoma drug development, just stays in the inhibition of tumor neovascularization^9^. Nevertheless, only regorafenib was approved as a second-line drug for intermediate or advanced hepatocellular carcinoma^10^. Therefore, it is necessary to explore new drug targets and develop different types of anti-hepatocarcinoma drugs for HCC treatment.

Currently, omic technologies have greatly promoted the findings of novel pharmaceuticals and drug targets^11,12^. During the past decade, major advancement in omic technologies (e.g., genomics, transcriptomic, proteomics, and metabolomics) had enabled high-throughput monitoring of a variety of molecular and organismal processes^13,14^. These techniques have been widely applied to identify biomarkers, characterize complex biochemical systems, study pathophysiological processes, map mechanisms of action and discover targets of novel drugs^14–18^. The cancer metabolome, as the complete set of small-molecule chemicals found within a biological sample, could be an important source for the discovery of molecular targets and mechanisms of action^19,20^. As a major carrier and functional executor of cellular activities, proteins own more biological information as compared to metabolites^21^. Over the last two decades, “proteomics” has emerged as a fascinating tool to probe the biological perturbations occurring and contribute more important insights into the action mechanisms of drug by a global analysis of protein alterations upon drug treatments^22,23^. Combining of multiple omic techniques is an emerging approach, which aims to help identify latent biological relationships^24^. Recently, integrated metabolomic and proteomic technologies have already been applied in the antitumor mechanism researches^25^.

As part of the effort in the discovery of potent anti-hepatocarcinoma agents, our laboratory has developed several series of novel compounds with obvious antitumor activity. Among them, a multi-substituted benzyl acridone derivative 8u had good activity against human liver carcinoma HepG2 cells and showed low toxicity *in vitr*o. Moreover, *in vivo* preliminary experiments showed that 8u might be a good lead compound in the treatment of HCC^26^. The results showed that 8u might have an anti-proliferative effect against human cancer cells through the induction of apoptosis. However, its possible molecular mechanism needs to be further improved, and its potential effect on cancer cell invasion and migration has never been observed before. In the current investigation, metabolomics and proteomics approaches were used to characterize alterations at the biochemical and molecular levels in control and 8u treated HepG2 cells. Furthermore, a series of bioassays were employed to in-depth discover the antitumor mechanism of 8u on HepG2 cells. This research revealed that acridone derivative 8u had the potential to develop into a new antitumor drug for HCC.

## Results and Discussion

### Differential metabolites identified and metabolism pathway analysis by LC/MS

**Figure 1:**
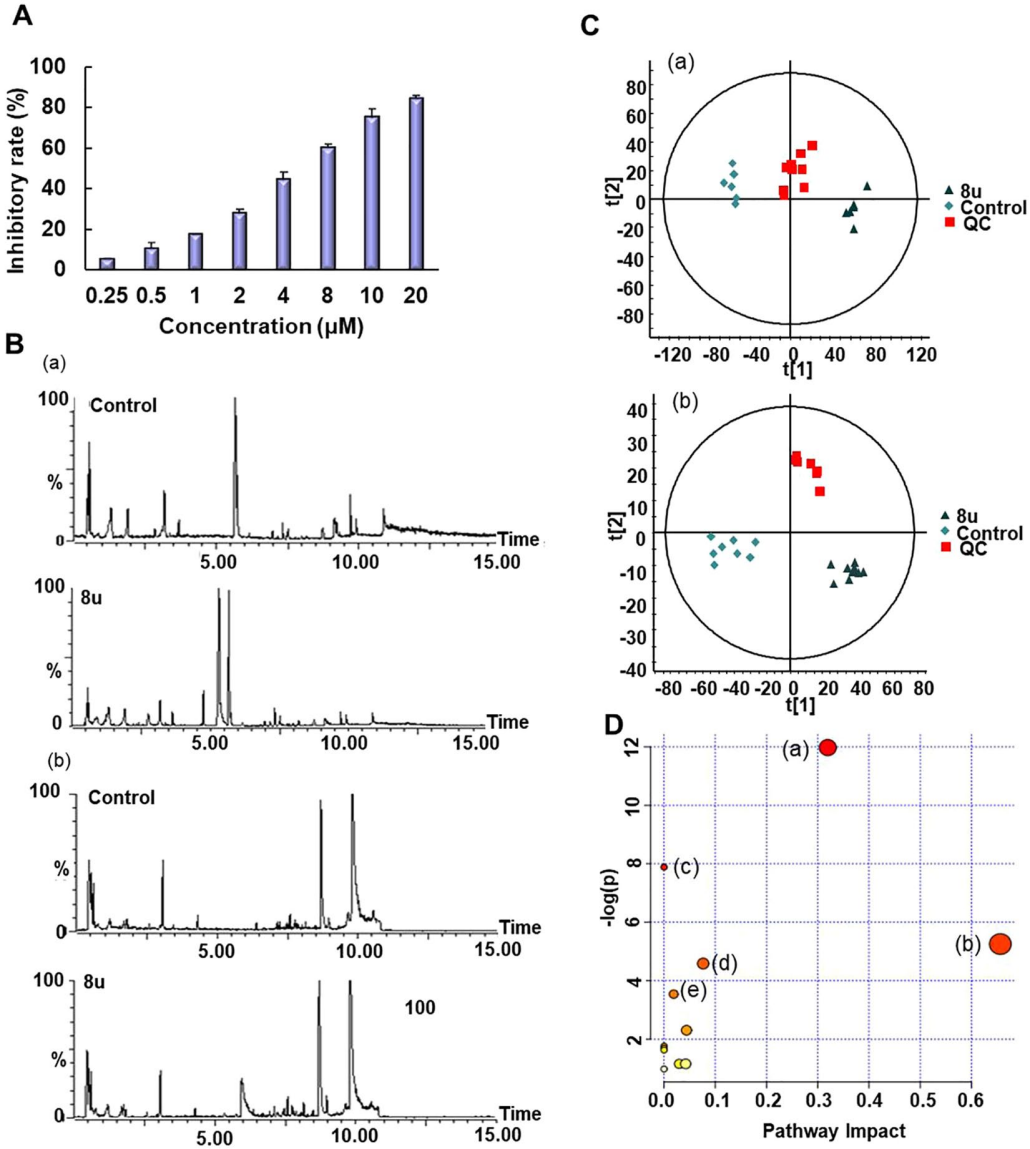
The influence of 8u on HepG2 cells metabolism. (**A**) The MTT assay of 8u treatment on HepG2 cells at 24 h. (**B**) The BPI chromatogram changes after treatment by 8u8u: (a) in the positive ion mode; (b) in the negative ion mode. (**C**) PCA between 8u and control groups: (a) in the positive ion mode; (b) in the negative ion mode. (**D**) Changes of metabolic pathways caused by 8u: (a) glycerophospholipid metabolism; (b) linoleic acid metabolism; (c) biosynthesis of fatty acids; (d) riboflavin metabolism; (e) purine metabolism.

Before the metabolomics experiment, the most optimal drug concentration was determined by MTT experiments. As shown in the Fig. 1A, the antiproliferative activity towards HepG2 cells increased significantly with increasing concentration of 8u. When the concentration increased to 2 μM, the inhibitor rate was approximately 20%. In this case, drug-induced changes could be observed, and sufficient cells could be ensured for subsequent detections. Thus, this concentration was appropriate for metabolomics study.

As Fig. 1B and C shown, 8u had a significant impact on metabolites in HepG2 cells. By differential analysis, 44 significantly changed metabolites were identified in positive and negative ion modes after 8u treated (Table S1). All of the altered metabolites were analyzed using KEGG database to identify the pathways involved. After searching against the database, the most abundant five pathways were presented in Fig. 1D. It could be easily observed that the differentially expressed metabolites were mainly involved in lipids metabolism (glycerophospholipid metabolism, linoleic acid metabolism, biosynthesis of fatty acids) and energy metabolism disorders (riboflavin metabolism and purine metabolism).

### 8u induced HepG2 cells apoptosis and cycle arrest

Lipids provide cancer cells with membrane structural units, signal molecules, protein post-translational modifications and energy supply to support rapid cell proliferation^27^. Hence, lipid metabolism disorders lead to the changes of membrane compositions, protein distribution and function, gene expression, and cellular functions, and then cause cell apoptosis^28^.

To determine the primary driver for the decreased cell viability, we analyzed the apoptosis of 8u treated HepG2 cells with flow cytometry. We observed a significant dose-dependent increase in the population of apoptotic cells in HepG2 cell lines when treated with 8u (Fig. S1). Apoptosis cell populations in HepG2 cells treated with 10 μM 8u for 48 h, were increased to 34.6% and 55.9%, respectively, compared to DMSO-treated control cells (0.039% and 0.405%). These results suggested that 8u could induce apoptosis to inhibit the proliferation of HepG2 cells.

Furthermore, we observed cell cycle arrest by 8u in HepG2 cells. Propidium iodide (PI) staining for HepG2 cell cycle progression showed that the majority of the cells were arrested in the S phase (Fig. S2A,B) after 8u treatment. Then, we examined the levels of several cell cycle regulators during the S transition. As the western blotting results shown, 8u treatment elevated levels of the CDK inhibitors p18, p21 and p27, decreased levels of CDK2, 4, 6 and cyclin A, B1, D3 in HepG2 cells compared with those in control cells (Fig. S2C,D). Taken together, our data demonstrated that 8u induced cell cycle arrest at S phase through upregulation of CDK inhibitors and downregulation of cyclin-dependent kinases (Cdks).

### 8u reduced migration and invasive capabilities of HepG2 cells

**Figure 2:**
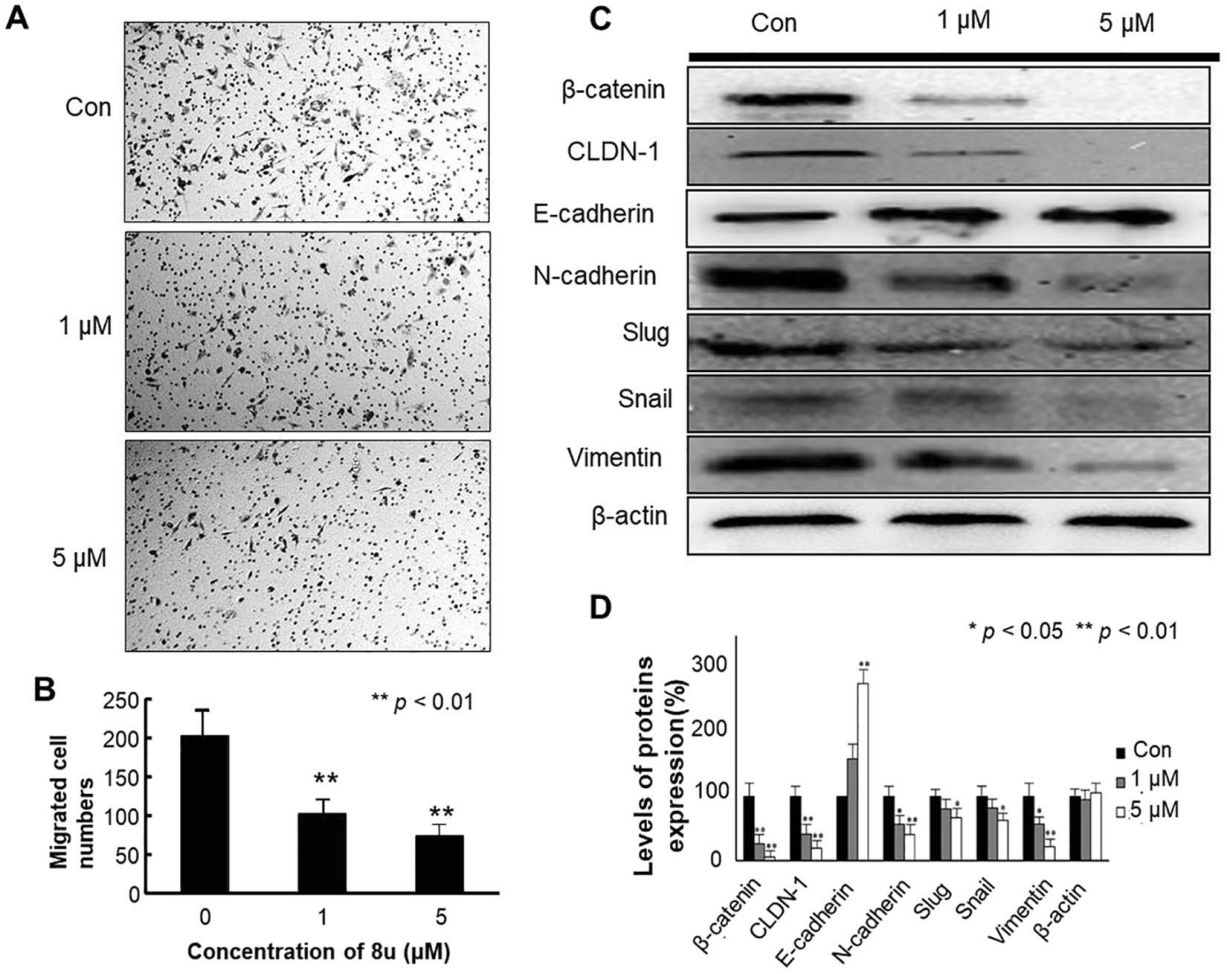
Effects of 8u on the migration and invasion capabilities in HepG2 cells. (**A**) Migration of HepG2 cells that underwent 8u treatment was determined in transwell invasion assay. HepG2 cells, after 6 h pretreatment with vehicle or indicated concentration of 8u, were added in the top chamber and allowed to invade for 24 h. Trypan blue-stained cells represent the fraction of cells that migrated from the top to the bottom chamber of the membrane. Three independent experiments were examined and representative images were presented. (**B**) Quantification of invasive HepG2 cells in the bottom chamber. (**C**) Expression levels of β-catenin, CLDN-1, E-cadherin, N-cadherin, Slug, Snail, Vimentin in cells treated for 48 h with 0, 1, 5 μM 8u. (**D**) The densitometry performed on the western blotting. Data are expressed as mean ± Standard Deviation (SD). Compared with the control group: *p < 0.05, **p < 0.01.

Lipid metabolism also plays a crucial role in cancer metastasis. During the progress of metastasis, lipid metabolism will change to facilitate the transfer of tumor cells^29^. Boyden chamber assay was employed to assess whether 8u could regulate HepG2 cell invasion. As shown in Fig. 2A and B, 8u significantly reduced the number of invasion cells stained with trypan blue compared to the control group. The result indicated that 8u could inhibit migration of HepG2 cells.

Western blotting assay was further employed to explore the anti-metastasis mechanism of 8u. Enhanced cell-to-cell junctions can reduce the invasive and metastatic abilities of cancer cells^30^. Hence, the expressions of cell-to-cell junctions related significant factors β-catenin, CLDN-1, E-cadherin, N-cadherin, Vimentin, Slug and Snail were detected. E-cadherin and Vimentin are essential proteins for homotypic adhesion junctions, which are the major markers of epithelial and mesenchymal cells, respectively^31,32^. Epithelial properties promote the adhesion junctions, and inhibit the invasion and metastasis of the tumor^33^. Western blotting results showed that the expression of E-cadherin was significantly up-regulated by 8u. On the contrary, expression of Vimentin was remarkably down-regulated in the 8u treated cells compared to control groups (Fig. 2C,D). The results showed that 8u could enhance epithelial properties of HepG2 cells.

E-cadherin and β-catenin can form complexes to promote cell adhesion^34^. When adherens junctions are disrupted, β-catenin could release from the complex and transfer into the nucleus, while transactively activating the expression of β-catenin gene^35^. N-cadherin is another type of cell adhesion molecule opposite to E-cadherin. It can stimulate migration and invasion of cancer cells^36^. Transcription factor Snail and Slug (snail 2) could suppress the expression of E-cadherin gene, further reduce cell-cell connections and enhance tumor cell invasiveness^33,37^. 8u also enhanced cell adhesion junctions by blocking the expressions of β-catenin and N-cadherin proteins as well as Snail and Slug transcription factors (Fig. 2C,D).

The claudin-1 (CLDN-1) is a key constituent of tight junctions (TJs), which is another mainly component for cell-to-cell adhesiveness^30^. Overexpression of CLDN-1 could promote invasion and metastasis of HCC cells through regulating the expression of N-cadherin, E-cadherin, β-catenin, Vimentin proteins and transcription factor Slug^38^. As the Fig. 2C,D shown, 8u repressed the expression of CLDN-1 protein, enhanced tight junctions and cell adhesion junctions.

For validating the regulation effect of 8u on invasive and metastasis, immunofluorescence analysis was performed using E-cadherin, β-catenin and vimentin antibodies (Fig. S3). The results were consistent with the western blotting experiments. Taken together, these results indicated that 8u reduced migration and invasion capabilities of HepG2 cells via enhancing the cell-cell contacts and making their epithelial properties more pronounced.

### 8u induced protein expression changes and inhibited the expression of HSP90α in HepG2 cells

**Figure 3:**
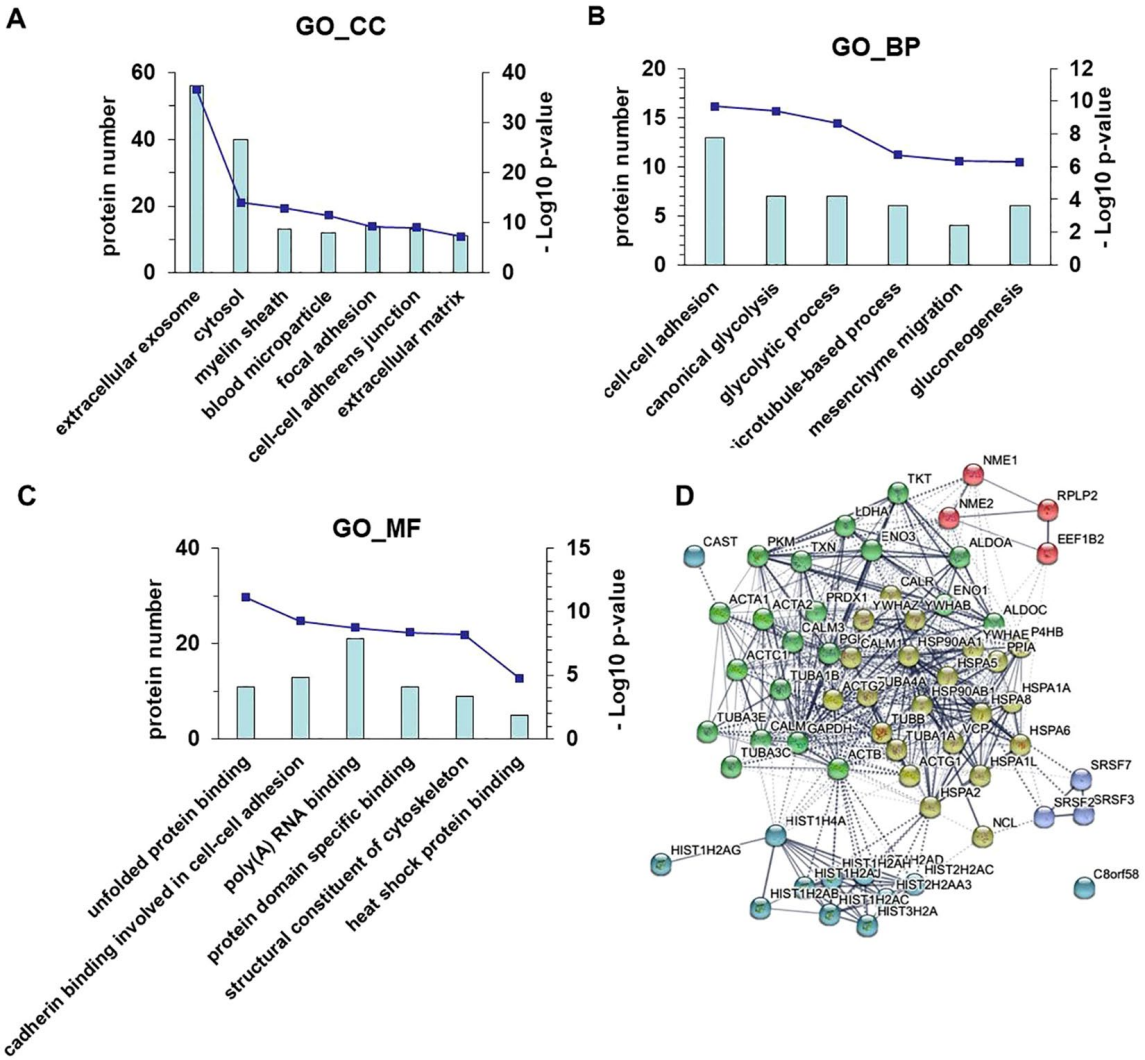
Functional annotation of significantly changed proteins by DAVID and STRING online database. Data show classes of proteins relative to (**A**) cellular composition, (**B**) biological processes, (**C**) molecular function. The bar graph shows the number of proteins in each gene ontology category, corresponding - log10 *p*-value. (**D**) protein-protein interaction analysis by STRING database. Different colors represent different clusters.

In order to comprehend the inhibition mechanism of 8u on invasion and metastasis, a proteomics approach was further performed to study the changes in proteins after 8u treated. A total of 137 differentially expressed proteins were discovered in the 8u treated group (Table S2). These proteins were functionally analyzed using the online DAVID, and were classified into three major categories (cellular component, molecular function, and biological process) according to GO analysis (Fig. 3).

Disruption of the cell-to-cell adhesiveness with changes in the expression of the corresponding junction proteins are closely related to cancer invasion and metastasis^30^. These proteins, which associated with cell-to-cell contacts, were dramatically changed after 8u treated. The cellular components results showed that these proteins were significantly enriched in broad GO FAT categories, including focal adhesion (p = 5.4 × 10^−10^), cell-cell adherens junction (p = 7.7 × 10^−10^), filopodium (p = 1.8 × 10^−3^) and microtubule (p = 4.1 × 10^−3^) (Fig. 3A). The biological processes showed that 8u treated group were mostly enriched in the GO FAT category of cell-cell adhesion (p = 2.1 × 10^−10^), microtubule-based process (p = 1.9 × 10^−7^), mesenchyme migration (p = 4.5 × 10^−7^), and heat shock protein binding (p = 3.8 × 10^−3^) (Fig. 3B). GO analysis for molecular functions showed that 8a-treated cells were mostly enriched in the GO FAT categories of cadherin binding involved in cell-cell adhesion (p = 5.3 × 10^−10^), structural constituent of cytoskeleton (p = 6.3 × 10^−9^), and heat shock protein binding (p = 1.7 × 10^−5^) (Fig. 3C).

These results validated our previous metabolic speculation, and were consistent with the results of transwell and western blotting experiments. To further identify the underlying protein complexes, we employed the STRING tool to generate a protein-protein interaction network and predict interactions between these proteins (Fig. 3D). The results showed that the HSP proteins family was the major linker protein. From proteomics data, 8u down-regulated the expression of HSP90α protein in HepG2 cells (Table S2).

### 8u could inhibit the expression of HSP90α protein and directly bind to it

**Figure 4:**
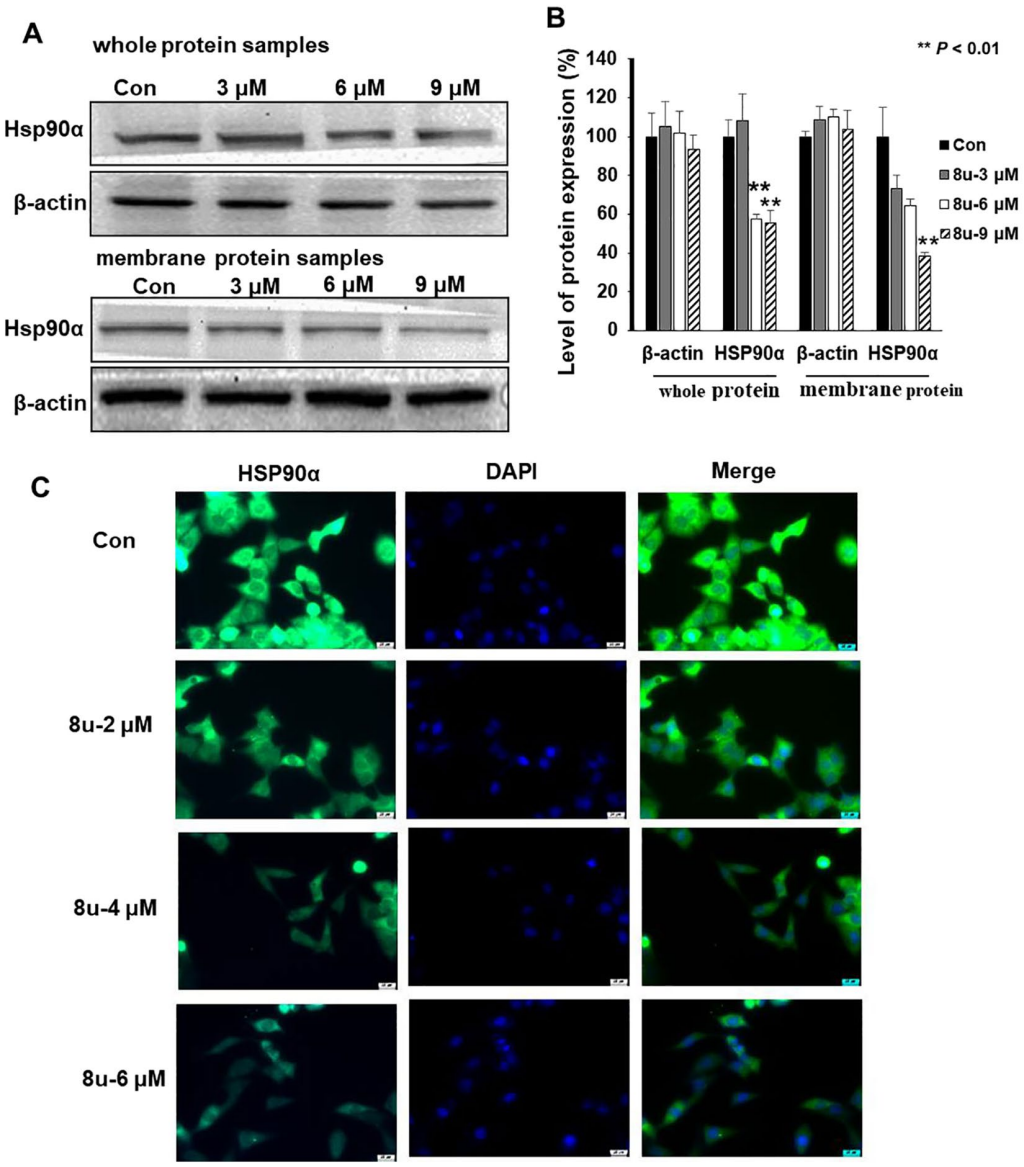
8u could inhibit the expression of HSP90α in HepG2 cells. (**A**) Western blotting analysis of HSP90α (whole and membrane samples) expression after cell exposure (or not) to 3, 6 and 9 μM of 8u for 24 h. (**B**) The densitometry performed on the western blotting. (**C**) Immunofluorescent analysis using Hsp90α Rabbit mAb (green). Blue were stained by DAPI for nucleus. Data are expressed as mean ± SD. Compared with the control group: *p < 0.05, **p < 0.01.

Depending on the results of proteomics, the changes of HSP90α protein expression were examined after 8u treated on HepG2 cells. 8u could significantly reduce the expression of HSP90α protein in whole cells and cell membrane (Fig. 4A,B). To validated the results, immunofluorescence experiments were employed. Figure 4C visually showed that 8u inhibited HSP90α protein expression in HepG2 cells in a dose-dependent manner.

**Figure 5:**
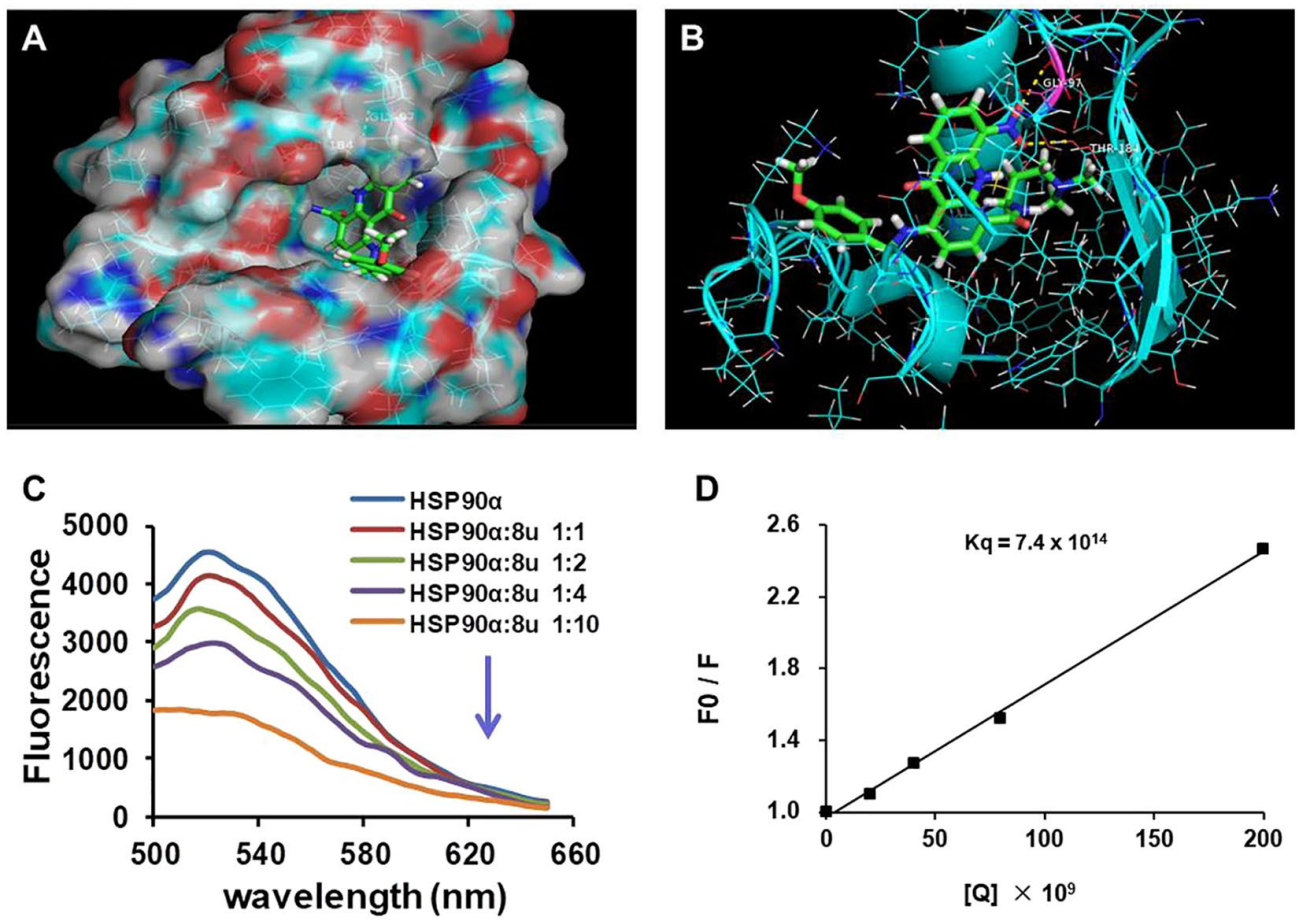
8u could directly bind to HSP90α protein. (**A**) Molecular docking model of compound 8u (stick and ball) binding to HSP90α protein using SYBYL-X v1.3 program. (**B**) Hydrogen bonds existed between 8u and amino acid residues of HSP90α (Gly97 and Thr184), Molecules were colored by atom type and hydrogen bonds were represented by yellow dotted lines. (**C**) The fluorescence was measured in the absence or presence of HSP90α, λ = 480 nm. The concentration of HSP90α was 20 nM. (**D**) The Sterne-Volmer quenching plots of the fluorescence titration. The quenching constant *k*_*q*_ is 7.4 × 10^14^ L·mol^−1^·s^−1^.

With the aim of observing whether 8u directly acted on HSP90α protein, the direct binding of 8u to HSP90α protein was assessed. First, molecular docking studies of the compound 8u with HSP90α model (PDB ID: 3TUH) were conducted using SYBYL-X v1.3 program. Ganetespib, an inhibitor of HSP90α, was as a control to analyze the binding sites of 8u and HSP90α protein. As seen from Fig. S4, four hydrogen bonds were formed between ganetespib and amino acid residues of HSP90α protein, two hydrogen bonds formed between the oxygen atom of carbonyl group of Ganetespib and the side chain of Asp93 and Lys58; another two formed between two nitrogen atom of triazole ring and the side chain of Gly97 and Thr184. It is notable that these amino acid residues (such as Gly97, Thr184, Asp93 and Lys58) constituted the key binding pocket for the HSP90α protein^39^. Similarly, 8u could also be well combined with HSP90α protein and formed two hydrogen bonds in the same binding pocket (Fig. 5A,B), one formed between the oxygen atom of nitro group and -COOH group of Gly97 (O···H-O); another formed between the oxygen atom of nitro group and -COOH group of Thr184 (O···H-O). Therefore, the docking results may suggest that the binding pocket of 8u and HSP90α protein is the same as Ganetespib.

To verify that 8u indeed binds to HSP90α, we performed a fluorogenic titration assay. Figure 5C showed that the fluorescence of HSP90α dramatically decreased in the presence of 8u. To confirm the binding affinity of 8u with HSP90α quantitatively, the classical Sterne-Volmer Equation (1) was utilized to calculate the binding constant^40^ (Fig. 5D).

1 $${F}_{0}/{F}=1+{K}_{sv}[{\rm{Q}}]\,=1+{k}_{q}{\tau }_{0}[Q]$$

*F*_0_ and *F* are the fluorescence intensities of HSP90α in the absence and presence of various concentrations of 8u. [Q] is the 8u concentration. *K*_*SV*_ is the Stern Volmer constant (quenching constant). τ_0_ is the average fluorescence lifetime of fluorophore in the absence of quencher (τ_0 = _10^−8^). *k*_*q*_ is the apparent biomolecular quenching constant which equals to *K*_*sv*_/τ_0_. The quenching constant *k*_*q*_ of 8u was 7.4 × 10^14^ L M^−1^ s^−1^. This value is three times higher than the quenching constant *(k*_*q*_ = 2.0 × 10^10^ L M^−1^ s^−1^) for the diffusion of the various quenchers in the solution^41^. This illustrated that the quenching effect of 8u on HSP90α was due to the static quenching caused by the formation of complexes. These analyses suggested that 8u might bind with HSP90α to contribute or partly contribute to its ability to inhibit tumor invasion and metastasis.

### 8u inhibited migration and invason by regulating the expression of HSP90α

**Figure 6:**
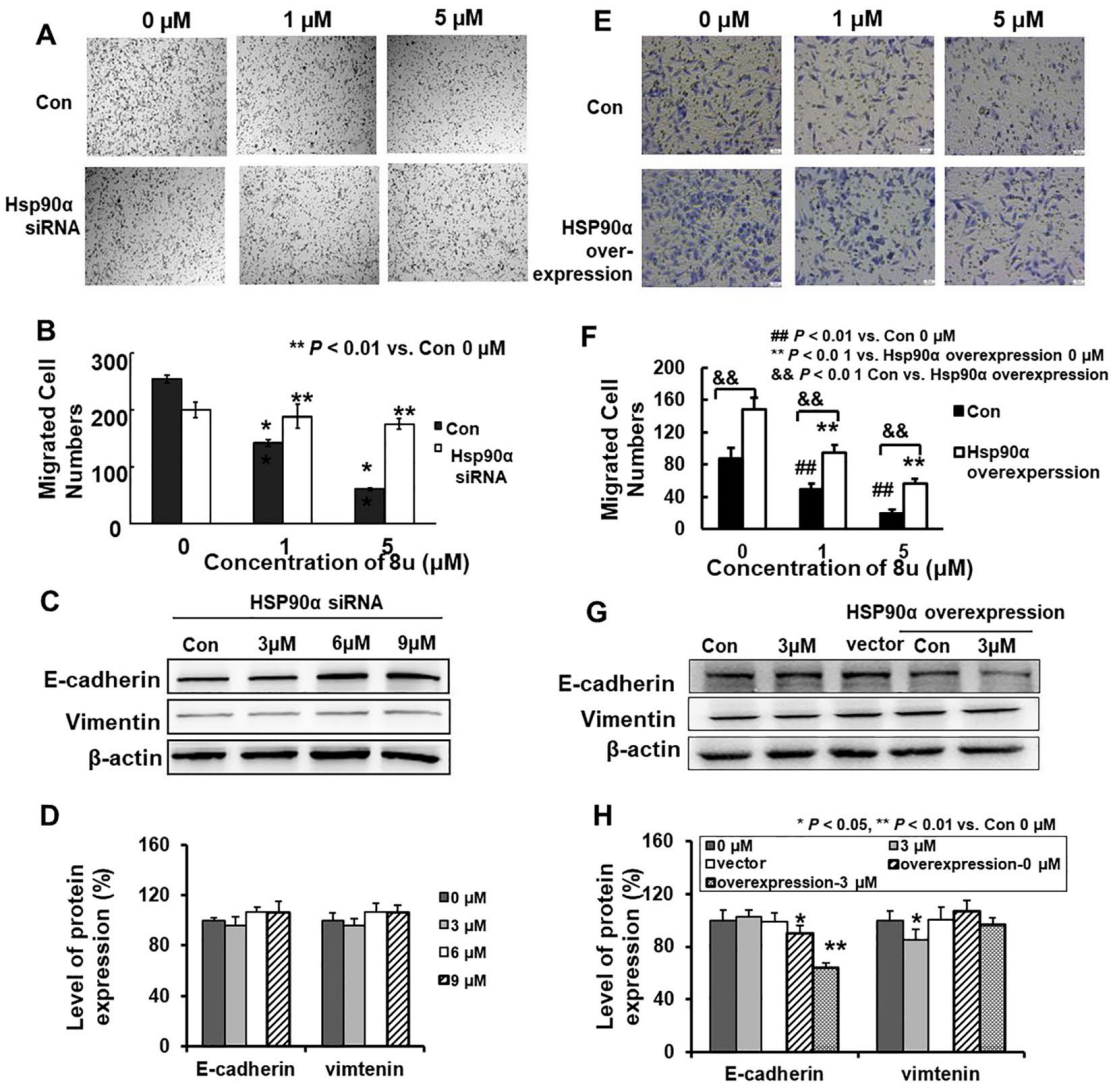
8u could regulate invasion and metastasis by inhibiting the expression of HSP90α in HepG2 cells. (**A**) After silencing HSP90α protein, the migration of HepG2 cells that underwent 8u treatment was determined in transwell invasion assay. Cells were transfected using 16 µl siRNA-Mate reagent and 100 nM HSP90α-siRNA. When the cells were transfected 48 h, transwell invasion assay was employed, which the protocol was same as Fig. 2A. Three independent experiments were examined and representative images were presented. (**B**) Quantification of invasive HepG2 cells in the bottom chamber. The statistical difference between the results was expressed as *p*-value. (**C**) After silencing HSP90α protein, the expression of E-cadherin and Vimentin treated with 0, 3, 6, 9 μM 8u for 24 h were determined by western blotting. (**D**) The densitometry performed on the western blotting of H. (**E**) After overexpression HSP90α protein, the migration of HepG2 cells that underwent 8u treatment was determined in transwell invasion assay. (**F**) Quantification of invasive HepG2 cells in the bottom chamber. (**G**) After overexpression HSP90α protein, the expression of E-cadherin and Vimentin treated with 0, 3, 6, 9 μM 8u for 24 h were determined by western blotting. (**H**) The densitometry performed on the western blotting of G. Data are expressed as mean ± SD. Compared with the control group: *p < 0.05, **p < 0.01.

Early research had shown that HSP90α was closely related to tumor invasion and metastasis^42^. Secretory HSP90α could promote tumor cell invasion^43^. However, the regulation of intracellular HSP90α on invasion and metastasis is unclear. Transwell invasion assay were used to observe the migration ability of HepG2 cells after HSP90α protein silencing. The invasive ability of HepG2 cells gradually weakened, with the increase of 8u dose. Under action of 1 μM 8u, the numbers of HepG2 cells were significantly reduced. However, after the silencing of HSP90α, this phenomenon disappeared, even if the dose increased to 5 μM, 8u could not prevent the invasion and metastasis of HepG2 cells (Fig. 6A,B).

Furthermore, the expressions of invasion and metastasis-related proteins in HepG2 cells were detected after silencing of HSP90α protein. In order to obtain a better effect of 8u, and a shorter acting time, we chose 3, 6, 9 μM 8u and 24 h for the subsequent Western blotting assays according to the IC_50_ value (Table S3). The results showed that the expression of E-cadherin significantly up-regulated, on the contrary, the expressions of β-catenin, CLDN-1, N-cadherin, Vimentin, Slug and Snail remarkably down-regulated in the silencing HSP90α group compared to negative group (Fig. S5). However, the regulation of 8u on E-cadherin and Vimtenin was weakened or even disappeared after silencing HSP90α (Fig. 6C,D). Therefore, the invasion of HepG2 cells in the transwell experiment was not significantly affected.

For purpose of confirming that 8u inhibited HepG2 cells invasion and metastasis by regulating HSP90α protein, an HSP90α expression plasmid was transfected into HepG2 cells. After 12 h, cells were divided into two parts, which one was used for transwell experiments and the other for western blotting experiments. HSP90α overexpression resulted in higher numbers of migrated cells than controls and attenuated the migration inhibition of 8u on HepG2 cell (Fig. 6E,F). For western blotting assay, cell lysates were collected and analyzed using HSP90α, E-cadherin and Vimtenin antibodies to examine the effects of 8u on cell-to-cell junctions. As the Fig. 6G and H shown, HSP90α overexpression could decrease the expression level of E-cadherin and prmote Vimtenin. In additional, HSP90α overexpression could reverse the inhibitory effect of 8u on Vimtenin. And the change of Vimtenin protein is positive correlated with HSP90α (Fig. S6). These were consistent with transwell experimental results. But E-cadherin was further inhibited after HSP90α overexpression. This may be related to the apoptosis induced by 8u, while apoptosis could cleave and shed E-cadherin^44^. These results indicated that 8u inhibited the invasion and metastasis of HepG2 cells by governing the expression of HSP90α protein.

### 8u could inhibit the activation of PI3K/Akt pathway

**Figure 7:**
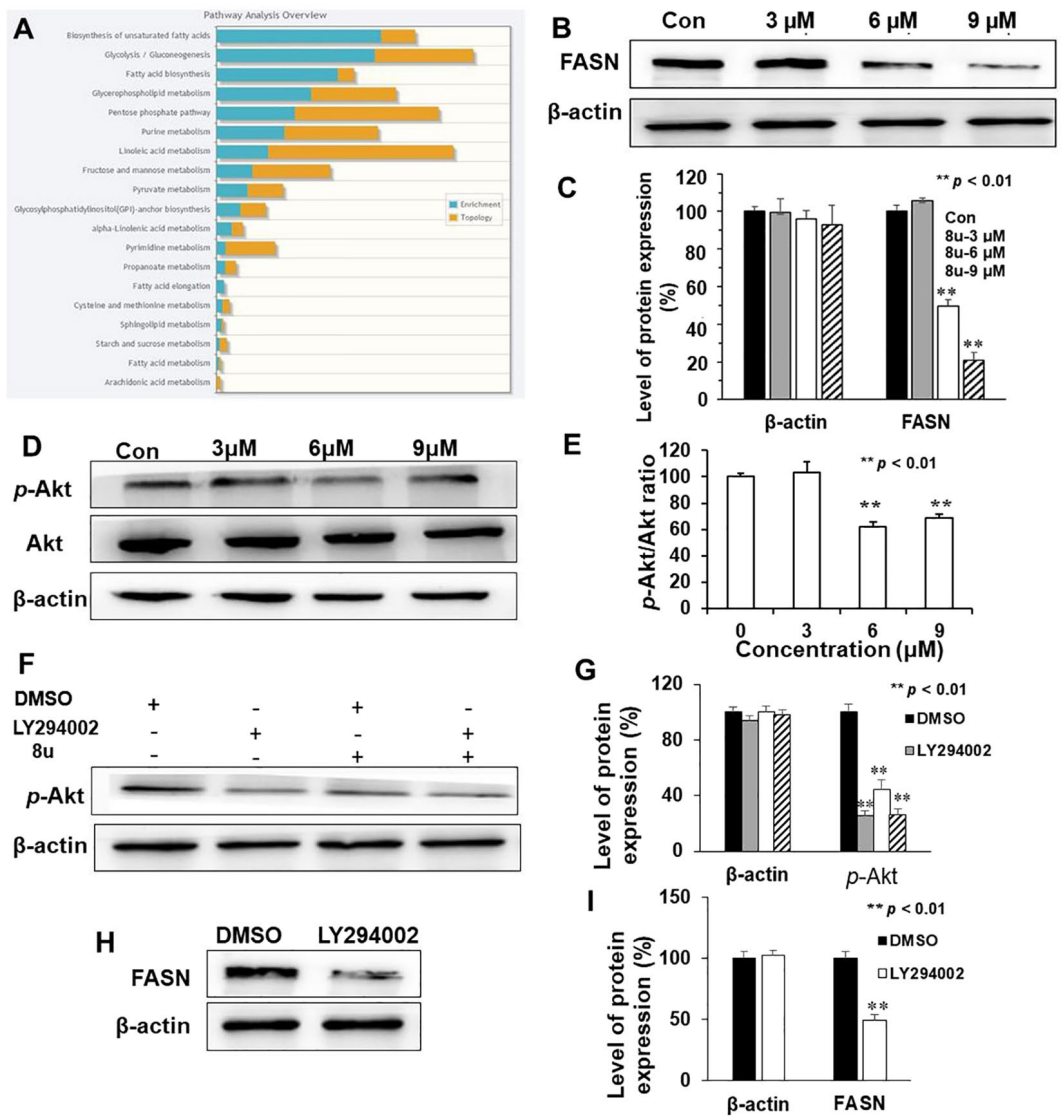
8u could inhibit the expression of FASN and inactivation of the PI3K/Akt pathway. (**A**) Integrated pathway analysis using Metaboanalyst. 8u mainly affected fatty acid biosynthesis. (**B**) Western blotting analysis of FASN protein expression after cell exposure (or not) to the shown concentrations of 8u for 48 h. (**C**) The densitometry of FASN protein performed on the western blotting of B. (**D**) Cells were treated with 8u at indicated concentrations for 24 h, and the expression of *p*-Akt and Akt proteins were determined by western blotting. (**E**) Quantification of *p*-Akt/Akt ratio were performance according to the western blotting results. (**F**) HepG2 Cells were pretreated for 2 h with or without 20 μM LY294002 and then with 8u (6 μM) for an additional 24 h. The phosphorylation of AKT was measured by western blotting. (**G**) The densitometry of *p*-Akt protein performed on the western blotting of F. (**H**) HepG2 cells were pretreated for 2 h with or without 20 μM LY294002 and then with DMSO for an additional 24 h. The FASN protein was measured by western blotting. (**I**) Densitometry of FASN protein performed on the western blotting of H. All the western blotting data presented were means ± SD of three independent experiments, and the significant difference was set at *p* < 0.05. *p < 0.05, **p < 0.01 compared with the control group.

To gain insight into how 8u might affect invasion and metastasis of HepG2 cells, integrated pathway analysis (IPA) was performed using Metaboanalyst. This analysis performed integrated metabolic pathway analysis on results obtained from the combined metabolomics and proteins expression. Our results showed that 8u mainly caused changes in biosynthesis of lipid, such as unsaturated fatty acids and fatty acid biosynthesis, and glycerophosphoipid metabolism (Fig. 7A), which were commonly found to enhance in HCC^45^.

FASN is a critical polypeptide enzyme for producing saturated fatty acids^46^. It can further influence the lipid metabolism^47^. When inhibiting metastasis, intracellular FASN protein expression is significantly decreased^48^. Thus, the effect of 8u on expression of FASN protein was examined. As the Fig. 7B and C shown, 8u significantly inhibited the expression of FASN protein in HepG2 cells.

PI3K/Akt pathway is a key signal pathway to regulate the expression of FASN protein and cancer invasion and metastasis^49,50^. The phosphorylation of Akt represents the activation of the PI3K/Akt pathway. In this study, the inhibitory effect of 8u on Akt phosphorylation was analyzed using western blotting analysis. As shown in Fig. 7D and E, 6–9 μM 8u decreased the levels of phosphorylated Akt, while the total Akt protein levels remained constant. Next, LY294002, a PI3K inhibitor, was used to determine whether 8u inhibited invasion and metastasis were associated with the PI3K/Akt pathway. As seen from Fig. 7F and G, 6 μM 8u significantly decreased Akt phosphorylation. Changes of FASN protein after LY294002 treatment were also examined. The result showed that the expression of FASN protein also reduced after inhibiting the activity of the PI3K/Akt signaling pathway (Fig. 7H,I). These results suggest that 8u can inhibit the activation of the PI3K/Akt signal pathway and further inhibit the expression of FASN protein.

### Activity of PI3K/Akt pathway interacts with the expression of HSP90α protein

**Figure 8:**
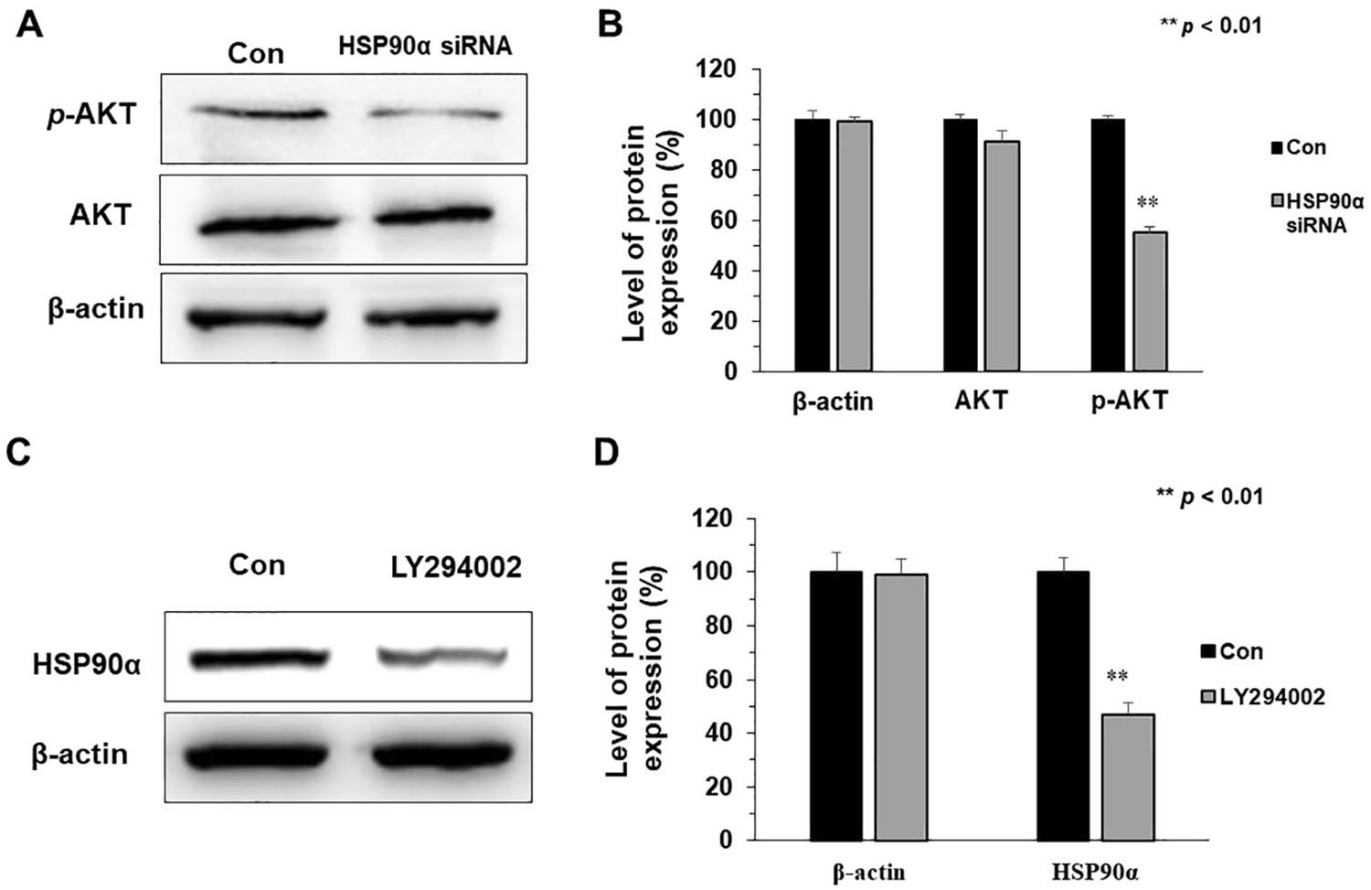
Activity of PI3K/Akt pathway interacts with the expression of HSP90α protein. (**A**) The expression of *p*-Akt and Akt proteins was determined by western blotting after silencing the HSP90α protein. (**B**) The densitometry performed on the western blotting of A. (**C**) The cells were treated with 20 μM LY294002 and the expression of HSP90α protein were determined by western blotting. (**D**) The densitometry performed on the western blotting of C. All the western blotting data presented were means ± SD of three independent experiments, and the significant difference was set at *p* < 0.05. *p < 0.05, **p < 0.01 compared with the control group.

In order to in-depth understand anti-metastasis mechanisms of 8u, the link between HSP90α protein and PI3K/Akt signaling pathway were explored. First, the expressions of *p*-Akt and Akt in HepG2 cells were examined after silencing HSP90α protein. As the Fig. 8A and B shown, phosphorylation of Akt protein in the HSP90α silencing group significantly reduced compared to the control group, whereas Akt content was not affected. We also found that overexpression HSP90α could upregulate the *p*-Akt expression level (Fig. S7). Next, expression changes of HSP90α after inhibiting the activity of the PI3K/Akt signaling pathway by LY294002. As shown in Fig. 8C and D, the expression of HSP90α protein significantly decreased after inhibiting of PI3K/Akt signal pathway. These results indicated that the activity of the PI3K/Akt pathway and the expression of HSP90α protein could co-adjust the cancer invasion and metastasis after 8u treated.

## Conclusion

**Figure 9:**
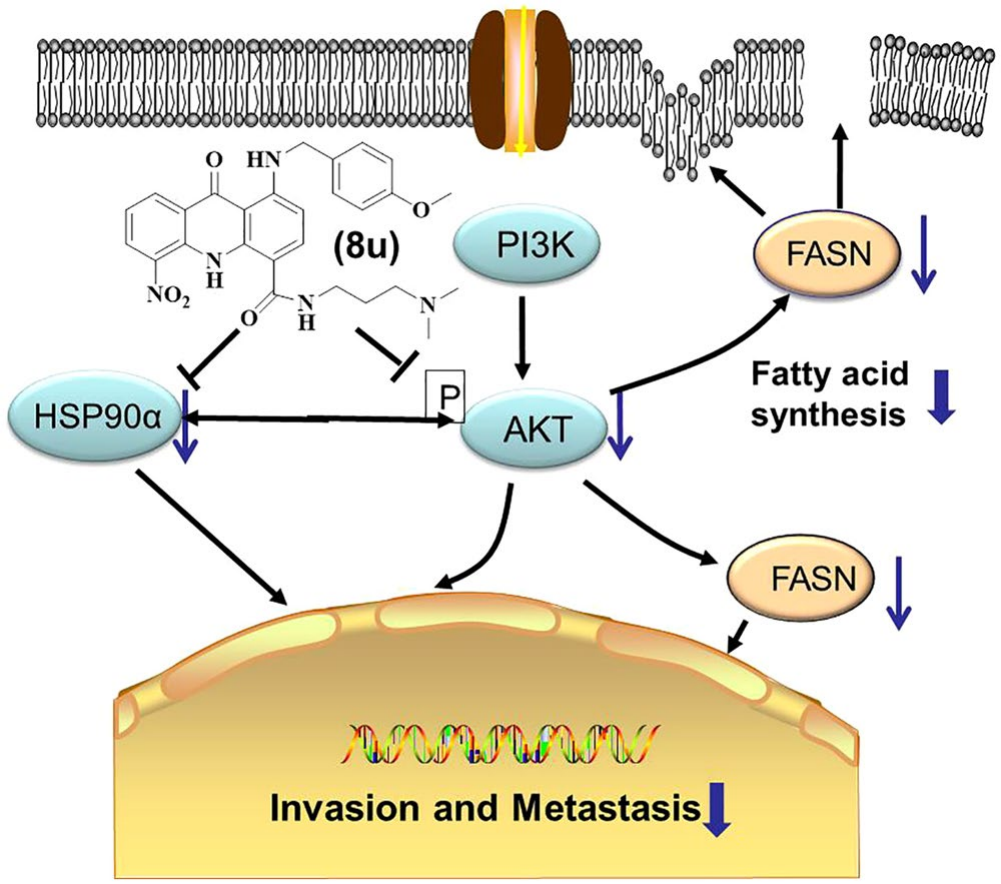
The schematic of the effect of 8u on invasive and metastasis. 8u inhibited the PI3K/Akt pathways and HSP90α protein to suppress cancer cell growth, invasion and metastasis. Blue arrows indicate downregulation. Black arrows indicate activation. Blunt ends indicate inhibition.

In summary, the novel acridone derivative 8u could inhibit the invasion and metastasis of HepG2 cells by enhancing cell-cell adhesion junctions. Mechanistically, we confirm that 8u could directly bind to HSP90α protein, and inhibit the migration and invasion by suppressing HSP90α. Furthermore, 8u also blocked PI3K/Akt pathway, thereby reducing FASN protein expression and disordering intracellular lipid metabolism to inhibit cell invasion and metastasis. Notably, suppression of PI3K/Akt pathway could down-regulate the expression level of HSP90α, and down-regulate of HSP90α could in turn inhibit the activity of PI3K/Akt pathway. The anti-metastasis mechanism of 8u on HCC was showed in Fig. 9. Taken together, this study demonstrates that 8u could inhibit invasion and metastasis of HepG2 cells by regulating the expression level of HSP90α protein and inhibiting the PI3K/Akt signaling pathway.

## Methods

### Cell culture and MTT assay

Detailed cell culture and MTT assay approaches were described in supporting information.

### Metabolomics analysis

HepG2 cells were exposed to 2 μM 8u, and were harvested by cold methanol/water in a ratio of 4:1 (v/v). The samples were analyzed by UPLC/Q-TOF MS. The chromatographic separation was carried out on a Waters AcquityTM BEH C_18_ column (100 mm × 2.1 mm, 1.7 μm). Raw UPLC-QTOF/MS ESI data were processed using the MarkerLynx software. Databases of HMDB (http://www.hmdb.ca/), Lipid Maps Database (http://www.lipidmaps.org) and METLIN (http://metlin.scripps.edu/) were used to identify the metabolite markers with tandem mass spectrometry. Some standards of metabolic interest were also used to confirm their structures.

### Label-free quantitative proteomics analysis

Cell culture and dosing conditions were the same as metabolomics methods. The proteins of cells were extracted and quantified. Then equal proteins (approximately 30 µg) were desalted and digested. The peptides mixture were desalted using C_18_ spin columns (Product # 89870, Thermo Scientific) and the flow-through was dried in a SpeedVac centrifugal evaporator. The dried peptides were dissolved in water for 2D LC-MS/MS analysis. A Waters system (Waters, Milford, MA) of 2D nano-Acquity UPLC coupled with Q-TOF premier tandem mass spectrometer was used for proteomics studies. Raw data from 2D nano-UPLC/Q-TOF were analyzed by the ProteinLynx Global Server (PLGS 2.5) with ExpressionE informatics. A Swiss-Prot database (release 51.0, October 2013) was used for database searches.

### Cell invasion assay

Cell invasion assay was performed as described^51,52^. Invasion inserts with 8μm pore membranes from Corning (New York, USA) were coated with fibronectin from Sigma-Aldrich (Missouri, USA) as described^53^. After treatment, cells were seeded on the inserts to reach confluence, and then culture for 24 hours with drug treatment. After fixed, the invading cells were stained with trypan blue, and were counted by Image-Pro-Plus 6.0 of Media Cybernetics (MD, USA).

### Western blotting analysis

Cell proteins were extracted by RIPA lysis as described^54^. Protein concentrations were measured by the Bradford protein assay. Equal amounts of protein were separated by 12% SDS polyacrylamide gel electrophoresis followed by transferring to PVDF membranes, and were subsequently analyzed with different antibodies. Specific-appropriate secondary antibodies were detected and measured by the Luminescence Image Analyzer Tanon 5200 (Shanghai, China). The density of the bands were measured by Image Quant software (Molecular Dynamics, Sunnyvale, CA, USA).

### Immunofluorescence staining analysis

8u treated cells were fixed with 4% paraformaldehyde for 25 min and permeabilized with 0.1% Triton X-100 for 10 min. Blocked with 2% bovine serum albumin (BSA) for 30 min at 37 °C and followed by the primary antibody against E-cadherin, β-catenin, Vimentin and HSP90α at 4 °C overnight. The cells were subsequently incubated with the corresponding Alexa 488-conjugated secondary antibody for 1 h at room temperature. The nuclei were stained with DAPI for 3 min. The images were captured using a DMI-4000B inverted fluorescence microscopy (Leica).

### Molecular bocking

Molecular modeling of small molecule compound 8u was performed with the molecular modeling package SYBYL-X 1.3 (Tripos associate Inc., St. Louis, MO, USA) according to the reported process^55^.

### HSP90α protein binding assay

The emission spectra were carried out on Fluorolog spectrometer. 3.6 µg purified HSP90α protein (StressMarq Biosciences Inc., Canada) was incubated in 2 ml buffer solution (100 mM Tris/HCl pH 7.5, 20 mM KCl, 6 mM MgCl_2_, 0.0005% BSA). The final concentration of the HSP90α was 20 nM. And then 8u8u was also added with the final concentration from 0 to 200 nM. The excitation wavelength was set at 480 nm. The ambient temperature of this experiment was maintained at room temperature. The incubation time before testing was 3 min.

### HSP90α siRNA transfection

Cells were transfected with HSP90α siRNA (sense: 5′-UCCGGUAUGAAAGTCUUGACTT-3′; anti-sense: 5′-GUCAAGCUUUCAUACCGGATT-3′) plus siRNA-Mate (Shanghai, GenePharma). Transfection assays was generated according to the manual’s protocol.

### HSP90α overexpression

HSP90α plasmid was constructed by genecreate (Wu Han, China). After ligation, the amplicon was transfected into puncture bacteria competent cells followed by plasmid extraction and transient transfection of HSP90α into HepG2 cells using polyplus as manual. After 12 h transfection, cells were replated and cultured for subsequent experiments.

## Electronic supplementary material

Supporting information
