# Supplementary material for: 8u, a pro-apoptosis/cell cycle arrest compound, suppresses invasion and metastasis through HSP90α downregulating and PI3K/Akt inactivation in hepatocellular carcinoma cells
Source: Sci Rep. 2018 Jan 10;8:309. doi: 10.1038/s41598-017-18701-3 (PMC5762664; doi:10.1038/s41598-017-18701-3)
Supplement: Supplementary file 1 — Supporting information [file 41598_2017_18701_MOESM1_ESM.doc]

**8u, a pro-apoptosis/cell cycle arrest compound, suppresses invasion and metastasis through HSP90α downregulating and PI3K/Akt inactivation in hepatocellular carcinoma cells**

Ning Wang^1,2,+^, Shaopeng Chen^2,+^, Bin Zhang^3^, Shangfu Li^2^, Feng Jin^4^,

Dan Gao^2,*^, Hongxia Liu^2^, Yuyang Jiang^2,5^

*^1^* *Department of Chemistry, Tsinghua University, Beijing 100084, China*

*^2^* *State Key Laboratory Breeding Base-Shenzhen Key Laboratory of Chemical Biology, Graduate School at Shenzhen, Tsinghua University, Shenzhen 518055, China*

*^3^ Li Dak Sum Yip Yio Chin Kenneth Li Marine Biopharmaceutical Research Center,*

*Ningbo University, Ningbo 315211, China*

*^4^ Neptunus Pharmaceutical Technology Center, Shenzhen, 518057, China*

*^5^ School of Medicine, Tsinghua University, Beijing 100084, China*

# Figures


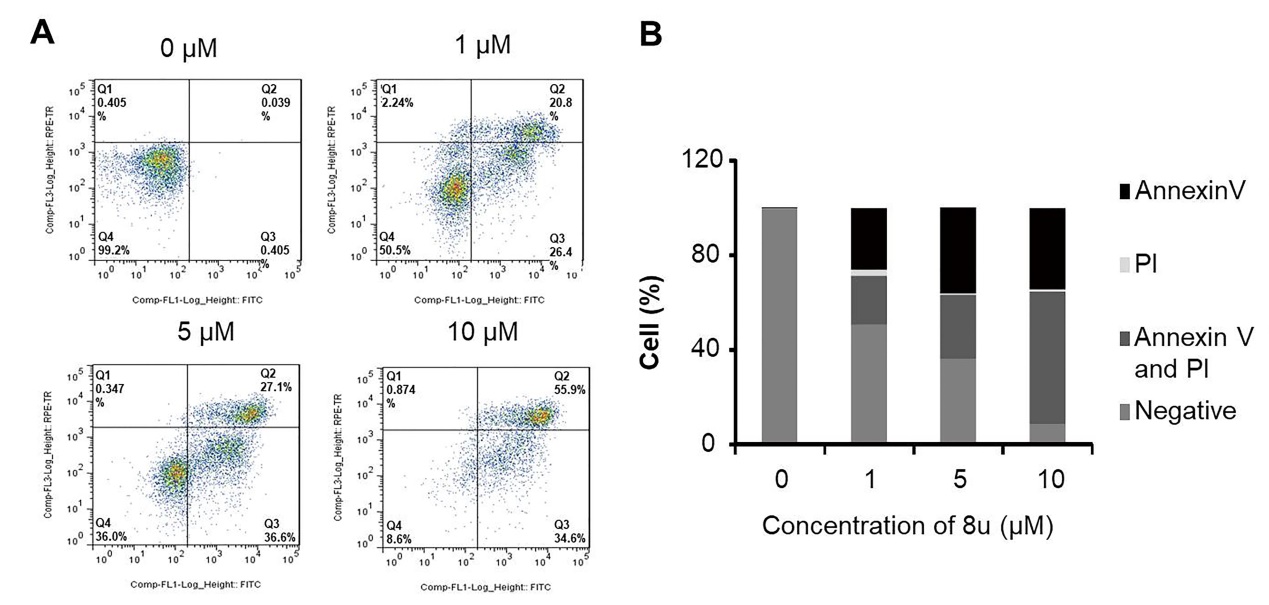


**Figure S1.** 8u induced apoptosis of HepG2 cells. (A) HepG2 cells were treated with 8u (0, 1, 5 and 10 μM) for 48 h and the cell apoptosis was evaluated by ﬂow cytometry after propidium iodide (PI) and Annexin V staining. The cell populations were discriminated in each quadrant as viable cells in the lower left (Q4, annexin V negative/PI negative), early apoptotic cells in the lower right (Q3, annexin V positive/PI negative), late apoptotic cells in the upper right (Q2, annexin V positive/PI positive), and necrotic cells in the upper left quadrant (Q1, annexin V negative/PI positive). (B) The quantitative data of panel A. The percentages of Annexin V-positive cells are shown

**
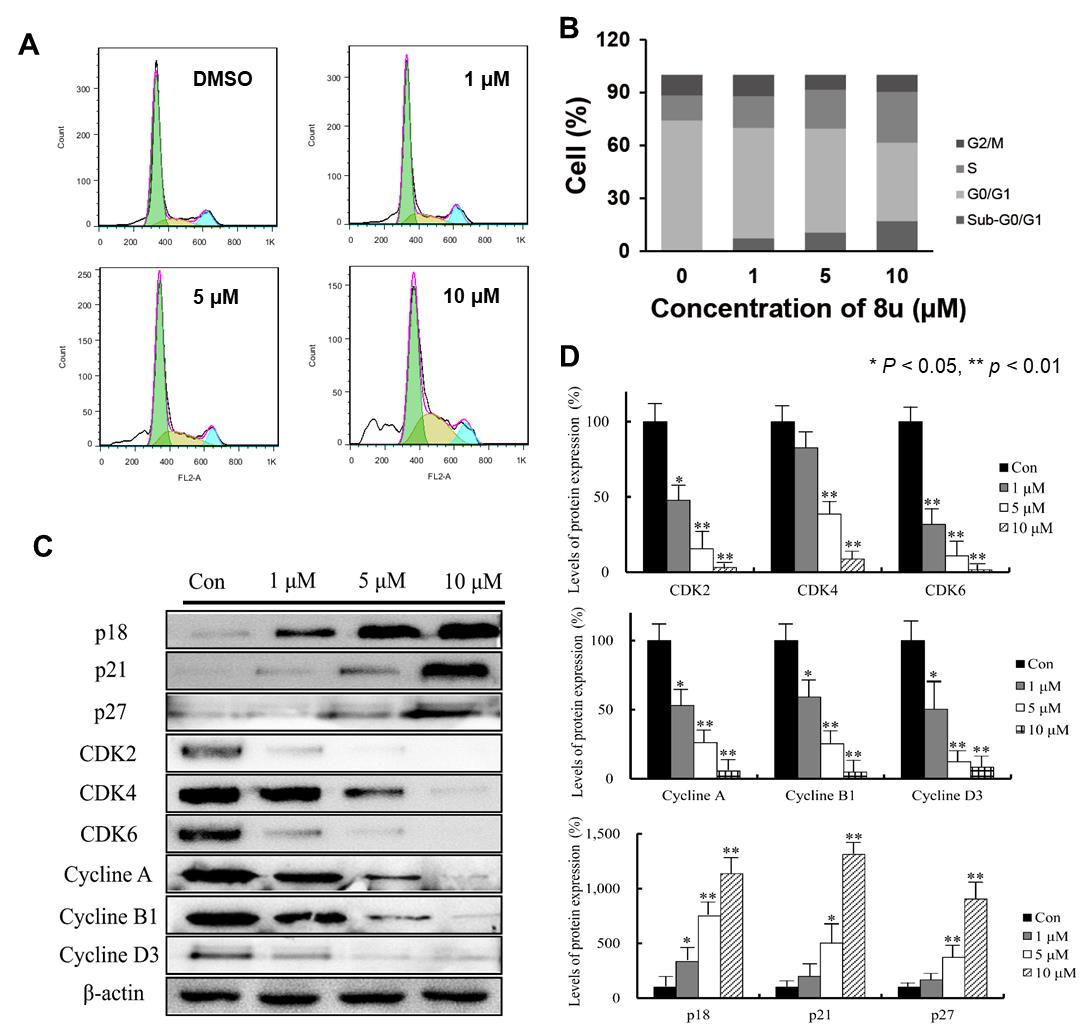
**

**Figure S2. 8u** arrested cell cycle progression at the S phase. (A**)** HepG2 cells were treated with 1, 5 and 10 μM 8u for 48h, and the cell cycle distribution was evaluated after propidium iodide (PI) staining. **(B)** Bar plot of the cell cycle distribution of HepG2 cells. (C) Expression of p18, p21, p27, CDK2, CDK4, CDK6, Cycline A, Cycline B1 and Cycline D3 proteins in HepG2 cells was determined by western blotting after 1, 5, 10 μM 8u treatment for 48h. Data are representative of three independent experiments. (D) The densitometry performed on the Western blotting.


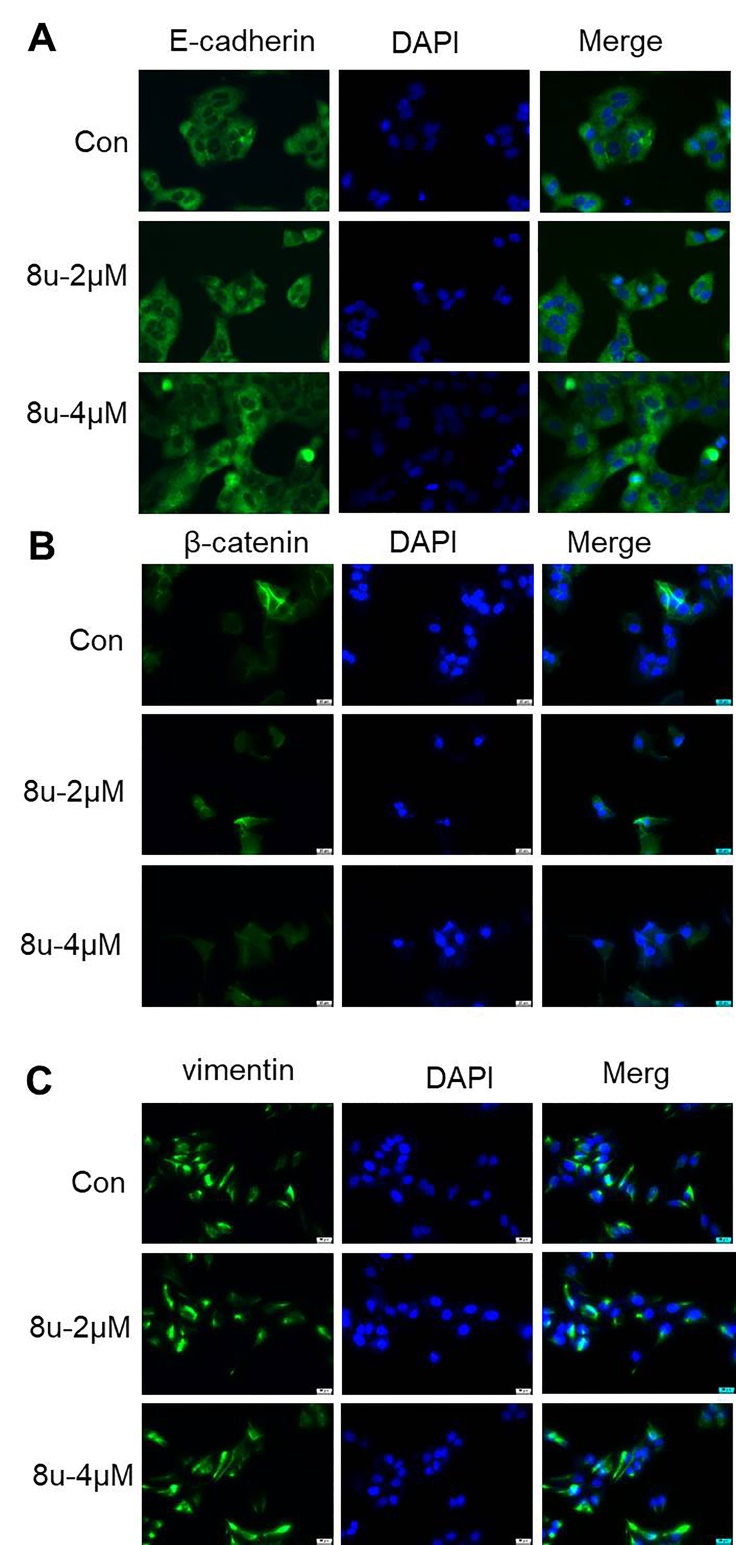


**Figure S3.** Eﬀects of 8u on the migration and invasion capabilities in HepG2 cells. (A) Immunofluorescent analysis using E-cadherin Rabbit mAb (green). Blue were stained by DAPI for nucleus. (B) Immunofluorescent analysis using β-Catenin Rabbit mAb (green). Blue was stained by DAPI for nucleus. (C) Immunofluorescent analysis using vimentin Rabbit mAb (green). Blue were stained by DAPI for nucleus. Bar equals 25μm.


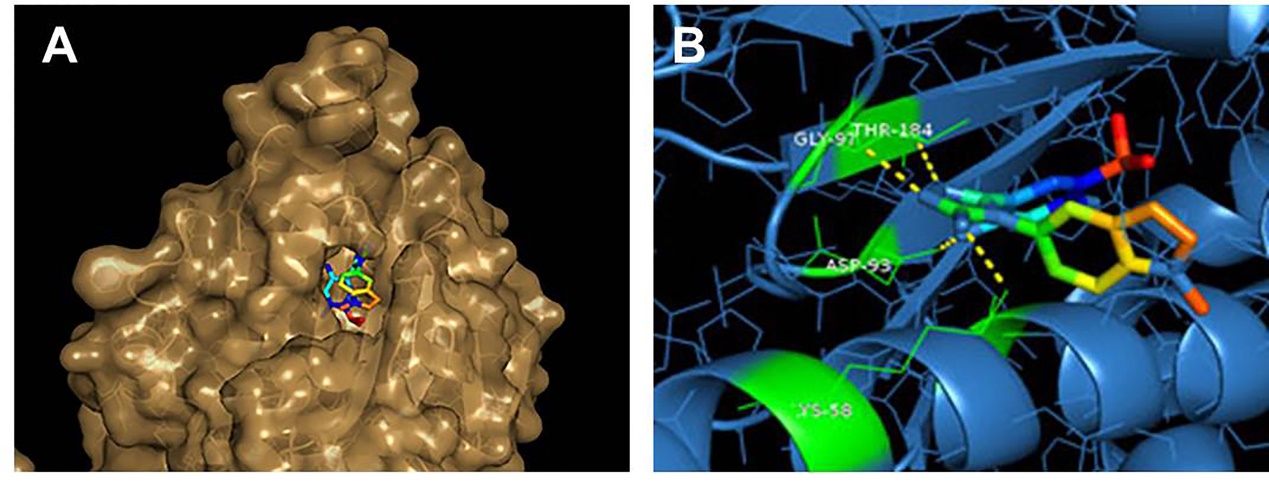


**Figure S4.** Molecular docking between HSP90α with ganetespib. (A) Molecular docking model of ganetespib (stick and ball) binding to HSP90α protein (marked with gold). (B) Hydrogen bonds existed between ganetespib and amino acid residues of HSP90α (Gly97, Thr184, Asp93 and Lys58), hydrogen bonds were represented by yellow dotted lines.


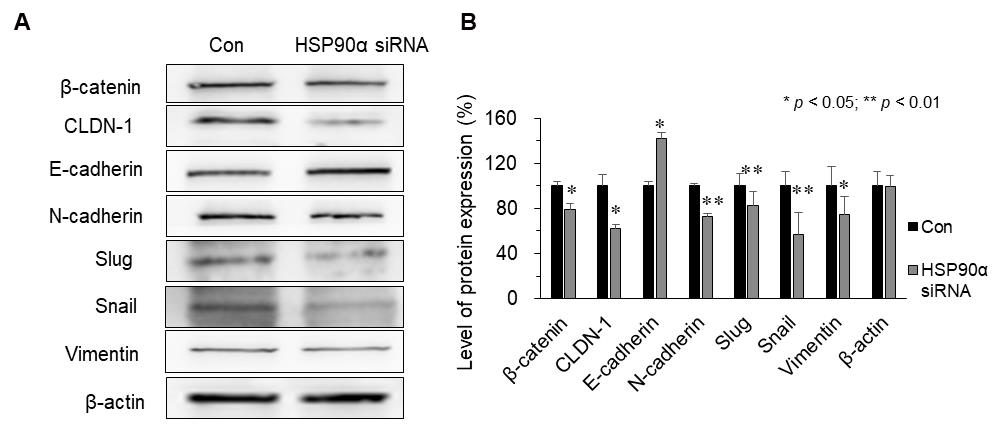
 **Figure S5.** Knockdown HSP90α could promote EMT process in HepG2 cells. (A) After silencing HSP90α protein, the expression of β-catenin, CLDN-1, E-cadherin, N-cadherin, Slug, Snail, Vimentin in cells were determined by western blotting. (B) The densitometry performed on the Western blotting.


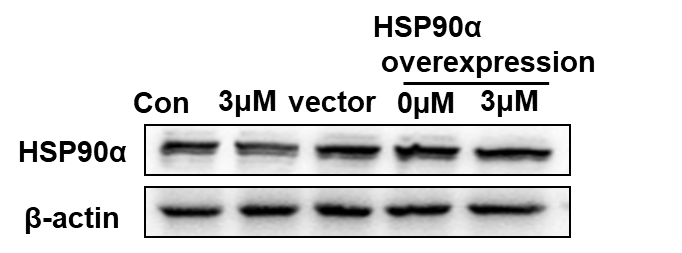


**Figure S6.** 8u regulated HSP90α expression after HSP90α overexpression.


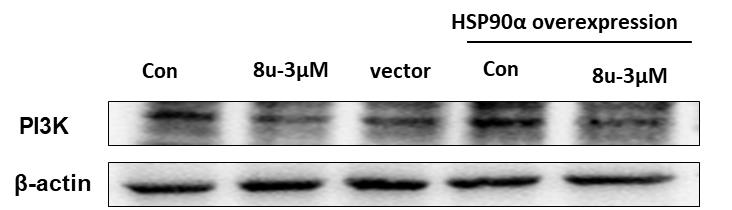


**Figure S7.** Overexpression HSP90α protein could prmote the expression of *p*-Akt.

# Tables

**Table S1.** **The significant changed metabolites by 8u on HepG2 cells.**

| **Compound** | **RT** | **m/z** | **△ppm** | **MS/MS fragments** | **Fold change^a^** |
| --- | --- | --- | --- | --- | --- |
| maltose | 0.5308 | 365.11 | 9 | 365.1; 203.0 | 0.56 |
| cyclic adenosine diphosphate ribose | 0.5442 | 540.06 | 2 | NA | 1.34 |
| Phosphocholine | 0.5499 | 184.08 | 23 | 184.0; 124.9; 86.0 | 0.76 |
| Adenine | 0.5565 | 136.07 | 34 | 119.0; 136.0 | 0.56 |
| 2-(hydroxyimino)- Pentanedioic acid | 0.5614 | 162.04 | 0 | 116.0; 84.0 | 0.69 |
| Adenosine | 0.5714 | 268.11 | 13 | 136.0 | 0.24 |
| Inosine | 0.5783 | 267.07 | 1 | 92.9 | 0.49 |
| Adenine | 0.581 | 134.05 | 9 | 134.0; 107.0 | 0.71 |
| 2-Hydroxy-6-oxo-(2'-aminophenyl)-hexa-2,4-dienoate | 0.581 | 232.06 | 8 | NA | 0.81 |
| PI(P-18:0/16:0) | 2.4295 | 823.59 | 18 | 823.5; 244.1; 129.0; 84.0 | 2.33 |
| PS(P-20:0/0:0) | 2.7237 | 560.33 | 3 | 560.3; 543.2 | 0.05 |
| LysoPC(18:2) | 2.7616 | 520.34 | 2 | 86.0; 184.0; 502.3; 520.3 | 0.19 |
| 5-Amino-6-(5'-phosphoribosylamino)uracil | 3.0598 | 353.05 | 10 | 353.0; 289.0; 273.0; 93.0; 80.96 | 0.71 |
| Lumichrome | 3.3676 | 243.09 | 16 | 243.08; 198.0; 172.0; 170.0; 145.0 | 0.72 |
| Pentadecylic acid(d3) | 4.7886 | 246.25 | 12 | 246.2; 228.2 | 0.41 |
| C16 Sphinganine | 5.6629 | 274.28 | 5 | 274.2; 256.2; 106.0; 88.0; 70.0 | 0.27 |
| Xestoaminol C | 5.7114 | 230.25 | 11 | 230.2; 212.2 | 0.37 |
| Phytosphingosine | 5.7252 | 318.29 | 17 | 318.2;300.2; 256.2; 102.0 | 0.31 |
| 16-hydroxy hexadecanoic acid | 6.7174 | 271.23 | 1 | 271.2; 225.2 | 1.74 |
| PI(16:0/0:0) | 6.9056 | 571.29 | 2 | 571.2; 255.2; 241.0; 153.0 | 30.84 |
| LysoPC(20:4) | 6.9571 | 544.34 | 7 | 544.3; 437.4; 341.6; 184.0; 104.1 | 0.33 |
| LysoPC(18:2) | 6.9669 | 520.34 | 3 | 520.3; 502.3; 337.2; 184.0; 104.1 | 0.56 |
| trans-Hexadec-2-enoyl carnitine | 7.0943 | 398.33 | 12 | 85.0; 144.1; 398.3 | 0.40 |
| PA(P-20:0/0:0) | 7.2903 | 485.28 | 1 | 485.2; 450.8; 96.7 | 1.60 |
| [Linoelaidyl carnitine](https://metlin.scripps.edu/metabo_info.php?molid=58412) | 7.3139 | 424.34 | 5 | 85.0; 102.0; 142.7; 221.0; 424.3 | 0.51 |
| Myristic acid | 7.3873 | 227.2 | 9 | standard | 1.74 |
| PI(18:0/0:0) | 7.7245 | 599.32 | 0 | 599.3; 419.2; 283.2; 241.0; 152.9 | 1.42 |
| PC(16:0/9:0(COOH)) | 7.9471 | 666.44 | 4 | 666.4; 184.0 | 3.90 |
| Pentadecylic acid | 8.051 | 241.22 | 1 | 241.2; 74.9 | 1.89 |
| Linoleic acid | 8.1492 | 279.23 | 3 | 279.2 | 1.64 |
| Palmitic acid | 8.7048 | 255.23 | 27 | 255.2 | 1.22 |
| Oleic Acid | 8.97 | 281.25 | 0 | 281.2 | 1.45 |
| PI(18:1/20:4) | 9.6929 | 883.54 | 1 | 883.5; 417.2; 303.2; 281.2 | 0.04 |
| Stearic acid | 9.8141 | 283.26 | 6 | standard | 1.13 |
| PI(18:2/18:1) | 9.9793 | 859.53 | 0 | 859.5; 435.2; 281.6 | 0.08 |
| PI(O-18:0/14:0) | 9.9893 | 795.56 | 24 | 795.5; 283.2 | 0.07 |
| PI(17:2/20:4) | 10.0197 | 867.52 | 22 | 867.4; 303.2; 146.9 | 0.35 |
| PG(18:1/16:0) | 10.1704 | 747.52 | 0 | 747.5; 281.2; 255.2 | 0.03 |
| PE(18:4/18:0) | 10.2278 | 738.51 | 1 | 738.5; 283.2 | 0.02 |
| PC(22:6/20:4) | 10.2352 | 852.58 | 32 | 852.5; 792.5; 303.2; 281.2 | 0.36 |
| PE(20:1/22:6) | 10.2393 | 816.57 | 13 | 816.5; 742.5; 279.2; 146.9 | 0.06 |
| PE(22:5/18:1) | 10.2716 | 790.56 | 19 | 790.5; 281.2; 255.2 | 0.03 |
| PE(22:4/P-18:1) | 10.5602 | 776.55 | 14 | 776.5; 478.2; 281.2;227.2 | 0.29 |
| PS(19:1/18:0) | 10.6779 | 802.56 | 4 | 744.5; 281.2; 255.2 | 0.13 |

Notes: a: 8u vs Control; NA: no MS/MS fragments.

**Table S2. Differentially expressed proteins in HepG2 cells treated with 8u.**

| **Accession^a^** | **Gene name** | **Protein. Description** | **Score^b^** | **Fold changes (8u /Control) ^c^** |
| --- | --- | --- | --- | --- |
| Q5T8M7 | ACTA1 | Actin alpha skeletal muscle | 744.84 | 0.32 |
| P68133 | ACTA1 | Actin alpha skeletal muscle | 1193.81 | 0.46 |
| A6NL76 | ACTA1 | Actin alpha skeletal muscle | 4022.55 | 0.66 |
| Q5T8M8 | ACTA1 | Actin alpha skeletal muscle | 4042.25 | 0.70 |
| P62736 | ACTA2 | Actin aortic smooth muscle | 1158.34 | 0.44 |
| F6UVQ4 | ACTA2 | Actin aortic smooth muscle Fragment | 2621.29 | 0.71 |
| F6QUT6 | ACTA2 | Actin aortic smooth muscle Fragment | 2621.29 | 0.72 |
| P60709 | ACTB | Actin cytoplasmic 1 | 1694.74 | 0.44 |
| E7EVS6 | ACTB | Actin cytoplasmic 1 Fragment | 712.07 | 0.46 |
| G5E9R0 | ACTB | Actin cytoplasmic 1 | 689.78 | 0.51 |
| P68032 | ACTC1 | Actin alpha cardiac muscle 1 | 1158.34 | 0.45 |
| P63261 | ACTG1 | Actin cytoplasmic 2 | 2478.29 | 0.43 |
| K7EM38 | ACTG1 | Actin cytoplasmic 2 N terminally processed Fragment | 689.78 | 0.46 |
| I3L4N8 | ACTG1 | Actin cytoplasmic 2 Fragment | 863.88 | 0.50 |
| I3L3R2 | ACTG1 | Actin cytoplasmic 2 N terminally processed Fragment | 712.07 | 0.52 |
| I3L1U9 | ACTG1 | Actin cytoplasmic 2 N terminally processed Fragment | 823.39 | 0.52 |
| J3KT65 | ACTG1 | Actin cytoplasmic 2 N terminally processed | 689.78 | 0.52 |
| I3L3I0 | ACTG1 | Actin cytoplasmic 2 N terminally processed Fragment | 823.39 | 0.53 |
| P63267-2 | ACTG2 | Isoform 2 of Actin gamma enteric smooth muscle | 709.36 | 0.33 |
| P63267 | ACTG2 | Actin gamma enteric smooth muscle | 1230.08 | 0.46 |
| B8ZZJ2 | ACTG2 | Actin gamma enteric smooth muscle | 3416.84 | 0.52 |
| F8WB63 | ACTG2 | Actin gamma enteric smooth muscle | 3416.84 | 0.53 |
| C9JFL5 | ACTG2 | Actin gamma enteric smooth muscle Fragment | 2621.29 | 0.72 |
| H3BR04 | ALDOA | Fructose bisphosphate aldolase A Fragment | 1085.54 | 0.67 |
| H3BU78 | ALDOA | Fructose bisphosphate aldolase Fragment | 1085.54 | 0.71 |
| P04075 | ALDOA | Fructose bisphosphate aldolase A | 3847.72 | 0.79 |
| J3KPS3 | ALDOA | Fructose bisphosphate aldolase | 2439.62 | 0.81 |
| K7EKH5 | ALDOC | Fructose bisphosphate aldolase C Fragment | 1794.21 | 0.45 |
| P09972 | ALDOC | Fructose bisphosphate aldolase C | 2567.34 | 0.48 |
| Q8NAV2 | C8orf58 | Uncharacterized protein C8orf58 | 376.35 | 15.18 |
| G3V479 | CALM1 | Calmodulin | 1659.4 | 0.36 |
| P62158 | CALM1 | Calmodulin | 1416.24 | 0.38 |
| H0Y7A7 | CALM2 | Calmodulin Fragment | 1309.73 | 0.44 |
| E7EMB3 | CALM2 | Calmodulin | 1309.73 | 0.45 |
| F8WBR5 | CALM2 | Calmodulin | 1416.24 | 0.50 |
| M0QZ52 | CALM3 | Calmodulin | 1416.24 | 0.39 |
| K7EL50 | CALR | Calreticulin Fragment | 1247.98 | 1.80 |
| H0Y9H6 | CAST | Calpastatin Fragment | 311.84 | 0.48 |
| P24534 | EEF1B2 | Elongation factor 1 beta | 2733.47 | 0.37 |
| F2Z2G2 | EEF1B2 | Elongation factor 1 beta | 2733.47 | 0.38 |
| C9JZW3 | EEF1B2 | Elongation factor 1 beta Fragment | 5885.04 | 0.40 |
| P06733-2 | ENO1 | Isoform MBP 1 of Alpha enolase | 2575.19 | 0.49 |
| P06733 | ENO1 | Alpha enolase | 1770.29 | 0.50 |
| P13929-2 | ENO3 | Isoform 2 of Beta enolase | 1257.09 | 2.01 |
| E7EUT5 | GAPDH | Glyceraldehyde 3 phosphate dehydrogenase | 3157.45 | 0.70 |
| P04908 | HIST1H2AB | Histone H2A type 1 B E | 1517.53 | 0.79 |
| Q93077 | HIST1H2AC | Histone H2A type 1 C | 1517.53 | 0.79 |
| P20671 | HIST1H2AD | Histone H2A type 1 D | 1517.53 | 0.79 |
| P0C0S8 | HIST1H2AG | Histone H2A type 1 OS Homo sapiens GN HIST1H2AG PE 1 SV 2 | 1517.53 | 0.78 |
| Q96KK5 | HIST1H2AH | Histone H2A type 1 H | 2292.89 | 0.79 |
| Q99878 | HIST1H2AJ | Histone H2A type 1 J | 1517.53 | 0.79 |
| P62805 | HIST1H4A | Histone H4 | 2196.3 | 2.77 |
| Q6FI13 | HIST2H2AA3 | Histone H2A type 2 A | 1517.53 | 0.79 |
| Q16777 | HIST2H2AC | Histone H2A type 2 C | 1517.53 | 0.79 |
| Q7L7L0 | HIST3H2A | Histone H2A type 3 | 1517.53 | 0.79 |
| P07900-2 | HSP90AA1 | Isoform 2 of Heat shock protein HSP 90 alpha | 1155.56 | 0.55 |
| P08238 | HSP90AB1 | Heat shock protein HSP 90 beta | 2018.68 | 0.53 |
| Q58FF7 | HSP90AB3P | Putative heat shock protein HSP 90 beta 3 | 795.29 | 0.52 |
| P08107-2 | HSPA1A | Isoform 2 of Heat shock 70 kDa protein 1A 1B | 646.62 | 0.52 |
| P08107 | HSPA1A | Heat shock 70 kDa protein 1A 1B | 646.62 | 0.61 |
| P34931 | HSPA1L | Heat shock 70 kDa protein 1 like | 634.35 | 0.39 |
| P54652 | HSPA2 | Heat shock related 70 kDa protein 2 | 759.07 | 0.45 |
| P11021 | HSPA5 | 78 kDa glucose regulated protein | 299.48 | 0.68 |
| P17066 | HSPA6 | Heat shock 70 kDa protein 6 | 704.27 | 0.27 |
| P48741 | HSPA7 | Putative heat shock 70 kDa protein 7 | 705.21 | 0.35 |
| E9PI65 | HSPA8 | Heat shock cognate 71 kDa protein Fragment | 669.33 | 0.31 |
| E9PN25 | HSPA8 | Heat shock cognate 71 kDa protein Fragment | 918.49 | 0.43 |
| E9PK54 | HSPA8 | Heat shock cognate 71 kDa protein Fragment | 979.21 | 0.44 |
| E9PQQ4 | HSPA8 | Heat shock cognate 71 kDa protein Fragment | 979.21 | 0.45 |
| E9PQK7 | HSPA8 | Heat shock cognate 71 kDa protein Fragment | 979.21 | 0.45 |
| E9PPY6 | HSPA8 | Heat shock cognate 71 kDa protein Fragment | 918.49 | 0.46 |
| P11142-2 | HSPA8 | Isoform 2 of Heat shock cognate 71 kDa protein | 1143.7 | 0.47 |
| E9PLF4 | HSPA8 | Heat shock cognate 71 kDa protein Fragment | 979.21 | 0.48 |
| E9PNE6 | HSPA8 | Heat shock cognate 71 kDa protein Fragment | 949.18 | 0.49 |
| E9PKE3 | HSPA8 | Heat shock cognate 71 kDa protein | 1082.99 | 0.51 |
| P11142 | HSPA8 | Heat shock cognate 71 kDa protein | 1463.51 | 0.53 |
| A8K7Q2 | HSPA8 | Heat shock cognate 71 kDa protein | 307.1 | 0.60 |
| P00338 | LDHA | L lactate dehydrogenase A chain | 7586.97 | 0.24 |
| F5GZQ4 | LDHA | L lactate dehydrogenase A chain Fragment | 4984.03 | 0.32 |
| F8W1N5 | NCAC | Nascent polypeptide associated complex subunit alpha muscle specific form Fragment OS Homo sapien | 2049.32 | 0.44 |
| H7BY16 | NCL | Nucleolin Fragment | 420.21 | 0.52 |
| P19338 | NCL | Nucleolin | 947.98 | 0.55 |
| P15531 | NME1 | Nucleoside diphosphate kinase A | 2017.36 | 0.59 |
| P15531-2 | NME1 | Isoform 2 of Nucleoside diphosphate kinase A | 2017.36 | 0.61 |
| Q32Q12 | NME1 | Nucleoside diphosphate kinase | 4600.9 | 0.61 |
| E7ERL0 | NME1 | Nucleoside diphosphate kinase A | 2017.36 | 0.61 |
| F6XY72 | NME1 | Nucleoside diphosphate kinase | 1783.84 | 0.63 |
| P22392 | NME2 | Nucleoside diphosphate kinase B | 2082.11 | 0.58 |
| J3KPD9 | NME2 | Nucleoside diphosphate kinase B | 2315.63 | 0.58 |
| P22392-2 | NME2 | Isoform 3 of Nucleoside diphosphate kinase B | 3971.9 | 0.61 |
| O60361 | NME2P1 | Putative nucleoside diphosphate kinase | 1848.6 | 0.60 |
| H7BZ94 | P4HB | Protein disulfide isomerase | 498.98 | 0.42 |
| P07237 | P4HB | Protein disulfide isomerase | 7860.76 | 0.44 |
| F5H8J2 | P4HB | Uncharacterized protein | 498.98 | 0.45 |
| B7Z7A9 | PGK1 | Phosphoglycerate kinase | 751.06 | 0.50 |
| P00558 | PGK1 | Phosphoglycerate kinase 1 | 972.19 | 0.54 |
| E7ERH5 | PGK1 | Phosphoglycerate kinase | 694.65 | 0.60 |
| H3BQ34 | PKM | Pyruvate kinase | 182.64 | 0.43 |
| H3BTN5 | PKM | Pyruvate kinase Fragment | 2381.91 | 0.53 |
| P14618 | PKM | Pyruvate kinase PKM | 2396.41 | 0.55 |
| P14618-3 | PKM | Isoform 3 of Pyruvate kinase PKM | 2279.88 | 0.57 |
| P14618-2 | PKM | Isoform M1 of Pyruvate kinase PKM | 3294.09 | 0.62 |
| Q504U3 | PKM2 | Pyruvate kinase | 2213.77 | 0.54 |
| P62937 | PPIA | Peptidyl prolyl cis trans isomerase A | 3143.68 | 0.63 |
| Q567Q0 | PPIA | Peptidyl prolyl cis trans isomerase A | 1871.98 | 0.64 |
| Q06830 | PRDX1 | Peroxiredoxin 1 | 928.9 | 0.57 |
| P05387 | RPLP2 | 60S acidic ribosomal protein P2 | 4581.91 | 0.58 |
| H0YDD8 | RPLP2 | 60S acidic ribosomal protein P2 Fragment | 5225.94 | 0.60 |
| B4DN89 | SFRS2 | Serine arginine rich splicing factor 2 | 783.31 | 0.56 |
| J3KP15 | SRSF2 | Serine arginine rich splicing factor 2 Fragment | 783.31 | 0.55 |
| J3QL05 | SRSF2 | Serine arginine rich splicing factor 2 | 1543.35 | 0.57 |
| Q01130 | SRSF2 | Serine arginine rich splicing factor 2 | 783.31 | 0.59 |
| P84103 | SRSF3 | Serine arginine rich splicing factor 3 | 865.37 | 0.67 |
| Q16629 | SRSF7 | Serine arginine rich splicing factor 7 | 916.95 | 0.05 |
| C9JAB2 | SRSF7 | Serine arginine rich splicing factor 7 | 1669.54 | 0.67 |
| Q16629-2 | SRSF7 | Isoform 2 of Serine arginine rich splicing factor 7 | 916.95 | 0.70 |
| P29401 | TKT | Transketolase | 1157.6 | 0.59 |
| P29401-2 | TKT | Isoform 2 of Transketolase | 874.96 | 0.60 |
| B4E022 | TKT | Transketolase | 824.16 | 0.62 |
| Q71U36 | TUBA1A | Tubulin alpha 1A chain | 500.78 | 0.58 |
| Q71U36-2 | TUBA1A | Isoform 2 of Tubulin alpha 1A chain | 544.43 | 0.59 |
| P68363 | TUBA1B | Tubulin alpha 1B chain | 500.78 | 0.58 |
| Q13748 | TUBA3C | OS Homo sapiens GN TUBA3C PE 1 SV 3 | 500.78 | 0.58 |
| Q13748-2 | TUBA3C | Isoform 2 of Tubulin alpha 3C D chain | 500.78 | 0.59 |
| Q6PEY2 | TUBA3E | Tubulin alpha 3E chain | 500.78 | 0.58 |
| P68366-2 | TUBA4A | Isoform 2 of Tubulin alpha 4A chain | 435.01 | 0.55 |
| P07437 | TUBB | Tubulin beta chain | 231.09 | 0.44 |
| P10599-2 | TXN | Isoform 2 of Thioredoxin | 2771.99 | 0.54 |
| P10599 | TXN | Thioredoxin | 2721.66 | 0.55 |
| P55072 | VCP | Transitional endoplasmic reticulum ATPase | 262.92 | 0.53 |
| P31946 | YWHAB | 14 3 3 protein beta alpha | 381.26 | 0.35 |
| P31946-2 | YWHAB | Isoform Short of 14 3 3 protein beta alpha | 276.67 | 0.36 |
| P62258 | YWHAE | 14 3 3 protein epsilon | 282.33 | 0.44 |
| H0YB80 | YWHAZ | 14 3 3 protein zeta delta Fragment | 1136.39 | 0.37 |
| B7Z2E6 | YWHAZ | 14 3 3 protein zeta delta | 1136.39 | 0.37 |
| P63104 | YWHAZ | 14 3 3 protein zeta delta | 1734.26 | 0.39 |
| P63104-2 | YWHAZ | Isoform 2 of 14 3 3 protein zeta delta | 1136.39 | 0.43 |

^a^ UniProtKB protein accession number.

^b^ PLGS score.

^c^ Only the proteins with a 40% cut-off for expression change were used in the analysis.

**Table S3. The IC_50_ values of 8u on HepG2 cells at different time.**

| **compound** | **IC_50_ (µM) -** HepG2 | |
| --- | --- | --- |
|  | 24h | 48h |
| 8u | 6.21 | 3.93 |

**Table S4. MTT screening of 8u on different cells**.

| **compound** | **IC_50_ (µM)** | | | | | | | |
| --- | --- | --- | --- | --- | --- | --- | --- | --- |
|  | QGY-7703 | MCF-7 ^a^ | T47D | MDA-MB-231 | HCT-116 | Hela | SK-OV-3 | U937 |
| 8u | 1.98 | 4.40 | >20 | 3.87 | 5.33 | 13.10 | 11.15 | 1.25 |

Note: a mean refers to reference 26. The effect time was 48h.

# Materials

## Reagents and materials

Compound 8u synthesized by our lab was solubilized in dimethyl sulfoxide (DMSO, Sigma-Aldrich Co., USA) at 2.5 mM stock. Acetonitrile and methanol were of HPLC grade and purchased from Fisher (Fairlawn, USA). HPLC grade formic acid was purchased from Tedia (Tedia Co., USA). Distilled water was filtered through a Milli-Q system (Millipore, USA). Dulbecco's Modified Eagle's Media (DMEM) and fetal bovine serum (FBS) were purchased from Gibco Corporation (Grand Island, NY). Penicillin-Streptomycin was obtained from North China Pharmaceutical, China. MTT (3-(4,5-dimethyl-2-thiazolyl)-2,5-diphenyl-2-H-tetrazolium bromide) was bought from Sigma (Sigma-Aldrich Co., USA). BCA Protein Assay Kit was purchased from Beyotime Institute of Biotechnology (Shanghai, China).

# Methods

## Cell culture and MTT assay

The human liver cancer cell line HepG2 was bought from the cell bank at the Chinese Academy of Sciences. Cells were grown in DMEM supplemented with 10 % FBS, 100 U/mL penicillin and 100 μg /mL streptomycin at 37 °C in an atmosphere of 5% CO_2_ in air atmosphere with the medium replaced every 24 h.

Cell viability was determined by the MTT assay as described ^1^. Cells (3×10^5^ cells/well) were treated with vehicle or different concentration of 8u for 24 h. A 10 μl of MTT (3-(4,5-dimethyl-thiazol-2-yl)-2,5-diphenyl-tetrazolium bromide from Sigma) solution (5 mg/mL) was added to each well in 190 μl of medium, and the plates were incubated for 4 h at 37 °C. 100 μl of DMSO were added to each well after removal of media. The absorbance was measured with Benchmark microplate reader (Molecular Devices Corporation, USA) at a wavelength of 490 nm. Percent viability was determined as the ratio between 8u-treated cells and untreated control cells, and IC_50_ was measured with Origin 75.

## Sample preparation for Metabolomics

HepG2 cells were exposed to 2 μM of 8u with equal amounts of DMSO as controls. Replicates in separate dishes for each group were analyzed. The final concentration of DMSO was less than 0.1%. After 24h incubation, cells were washed twice by 1.0 mL phosphate-buﬀered saline (PBS). Then we immediately added 1.0 mL mixture of methanol/water in a ratio of 4:1 (v/v) at -20 °C to harvest cells. The cell numbers were then counted with hemacytometer, which used for normalization of the metabolite levels. Then the cells were immediately transferred to an ice bath, ultra-sonicated for 10 min and subsequently centrifuged at 13000 g for 10 min at 4 °C. Supernatants were collected and dried with a stream of nitrogen. The residues were resuspended in 1.0 mL acetonitrile/water (1:1, v/v) mixture, and were filtered through 0.22 μm mesh millipore filters (Florham Park, NJ) into glass auto-samplers. The samples were stored at – 80 °C prior to analysis. In parallel, a quality control (QC) sample was prepared by mixing equal volumes of 30 mL into a glass auto-sampler from each of the samples. The pooled QC samples were injected three times at the beginning and of the run in order to condition or equilibrate the system and then analyzed every three samples to further monitor the stability of the analysis.

## UPLC/Q-TOF MS Conditions for metabolomics

UPLC/Q-TOF MS analysis was performed using Acquity TM Ultra-Performance Liquid Chromatography system (Waters Corporation, MA, USA) coupled to Q-TOF premier Mass Spectrometer (Waters Corporation, MA, USA). The chromatographic separation was carried out on a Waters AcquityTM BEH C18 column (100 mm x 2.1 mm, 1.7 μm). The column and samples were maintained at temperatures of 35 °C and 4 °C, respectively. The flow rate was at 0.5 mL/min. The mobile phase consisted of (A) 0.1% formic acid in water and (B) acetonitrile in positive mode, while (A1) 5 mM ammonium acetate in water and (B1) 5 mM ammonium acetate in acetonitrile (95:5, v/v) in negative mode. Elution gradient was maintained at 5% B for 1min, linearly increased from 5% to 40% B (B1) within 5 min, then to 95% B (B1) within 5 min and held for 1 min, followed by return to 5% B. Total running time was 15 min per separation. A 10 μL sample volume was introduced onto the column. The Waters Q-TOF premier equipped with an electrospray ion source in both positive and negative ion electrospray (ESI+ and ESI-) modes and V optics mode. The capillary voltage was 3.0 kV (ESI+) and 2.5 kV (ESI-), cone voltage was 30V (ESI+, ESI-). Cone gas flow was set at 50 L/hr with source temperature of 150 °C. Desolvation gas flow was maintained at 600 L/hr with the desolvation gas temperature of 300 °C. Data were collected in the centroid mode. The mass range was m/z 50 – 1000 with a scan time of 0.2 s and interscan time 0.02 s. For mass accuracy a LockSprayTM interface was used with Leucine-enkephalin (m/z 556.2771, ESI+; m/z 554.2615, ESI-) at a final concentration of 80 pg/uL in acetonitrile-water with 0.1% formic acid (50 : 50 v/v) was used as the lock mass (m/z 556.2771) with a flow rate of 0.05 mL/min. Lock spray frequency was set at 10 s and scan to average for correction was 10 s with the reference cone voltage at 35 V. For MS/MS analysis, the collision energies were set in ramp mode ranged from 10 V to 40 V.

## Data processing and multivariate statistical analysis

Raw UPLC-QTOF/MS ESI data were processed using the MarkerLynx software (version 4.1, Waters Corporation, MA, USA) which used Apex-Track-peak detection package to integrate peaks in UPLC/MS data. Peaks in each chromatogram were identified by m/z, retention time (RT) as well as their associated height intensities. The three-dimensional data, peak number (RT m/z pair), sample name, and normalized ion intensity were introduced to SIMCA-P 11.5 software package (Umetrics, Umea, Sweden) for multivariate data analysis. The variables were transformed by mean-centering and Pareto scaling to increase the low abundance ions without significant amplification of noise, and then analyzed by PCA, which is used for variable reduction and separation into classes. Databases of HMDB (<http://www.hmdb.ca/>), Lipid Maps Database (http://www.lipidmaps.org) and METLIN (http://metlin.scripps.edu/) were used to identify the metabolite markers with tandem mass spectrometry. Standards of metabolic interest were used to confirm their structures.

## Sample preparation for proteomics

Cell culture and dosing conditions were the same as metabolomics methods. Cells resuspended in lysis buffer combined with proteinase inhibitor cocktail (Thermo Scientific, USA). The total protein concentration was measured using Thermo Scientific Micro BCA Protein Assay kit according to the manufacture’s protocol. Then equal proteins (approximately 30 µg) were diluted with 450 µL 100 mM ammonium bicarbonate and added to the upper chamber of the spin filter with a 3000 molecular weight cutoff from Millipore (Billerica, MA, USA). The samples were centrifuged at 14,000 g to pass the solution through the filter. The proteins were then reduced with 10 mM DTT, alkylated with 20 mM iodoacetamide, and digested with 0.6 µg modified sequencing grade trypsin (Promega, Madison, WI, USA) dissolved in 100 mM ammonium bicarbonate. Above three procedures were performed using spin filter as the tryptic peptides were collected by centrifugation through the filter at 4500 g. The peptides mixture were desalted using C_18_ spin columns (Product # 89870, Thermo Scientific) in accordance with the manufacture’s protocol, and the flow-through was dried in a SpeedVac centrifugal evaporator. The dried peptides were dissolved in water for 2D LC-MS/MS analysis.

## 2D nano-UPLC/Q-TOF MS conditions

A Waters system (Waters, Milford, MA) of 2D nano-Acquity UPLC coupled with Q-TOF premier tandem mass spectrometer was used for proteomics studies. A combination of high pH reverse phase and conventional low-pH reverse phase served as a two-dimensional protein separation method. Solvents for the first-dimension LC of high-pH reverse phase were 20 mM ammonium formate, pH10, (solvent A), and 100% acetonitrile (solvent B), respectively. The online fractionation was carried out with a trap column and percentage of solvent B. The peptide sample was loaded onto an XBridge BEH130 C_18_ trap column (5 µm particle, 300 µm id, 50mm long, PN# 186003682; Waters). The partial-loop sample loading method was configured with a 10 µL sample loop in the autosampler for maximal loading with zero sample loss (5 µL for each injection). The fractionation and desalting were carried out using a 2D-dilution method configured within the 2D nano-Acquity and controlled with MassLynx 4.1 (Waters). There were five fractions based on percentage of solvent B as follows: (1) 10.8%; (2) 14.0%; (3) 16.7%; (4) 20.4%; (5) 60.5%.

For the second-dimension LC, a BEH130 C_18_ column (1.7 µm particle, 75 µm id, 200 mm long, PN# 186003546; Waters) was used for peptide separation. A Symmetry C_18_ (1.7 µm particle, 75 µm id, 200 mm long, PN# 186003514; Waters) served as a trap/guard column for desalting and pre-concentrating the peptides for each fraction. The solvent components consisted of (A) 0.1% formic acid in water and (B) 0.1% formic acid in acetonitrile. Elution gradient was as follows: 0 - 2 min, 2% B; at 2.5 min, 7% B; at 80 min, 45% B; at 85 min, 85% B; at 90 min, 85% B; at 90.1 min, 1% B; 90.1 - 95 min, 1% B. The nano-flow rate was 0.5 µL/min. A blank injection using the same LC method was introduced between each sample to remove carry overs or contaminants.

The parameters for the MS instrument were as follows: Nano-ESI+ selected as ionization source using the Nanosprayer (Waters) coupled with a PicoTips emitter (PN#FS360-20-10-CE-20, New Objective, Woburn, MA), capillary voltage set at 3 kV, sample cone voltage 30 V, extraction cone voltage 4.0 V, collision energy for MS1 scan 6 V, TOF reflectron operated in V-mode, source temperature set at 100 °C. The mass accuracy of the raw data was corrected using Leucineenkephalin (200 fmol/µL, 500 nL/min flow rate, 556.2771 Da [M+H]^+^) that was infused into the mass spectrometer as a lock mass during sample analysis, and all MS measurements were within 30 ppm.

Nano-LC-MS/MS was carried out for each fraction with a data-independent scanning (MSE) survey method. MSE experiments were automatic and planned to step between standard MS (6 eV) and elevated collision energies MSE (12 – 45 eV) applied to the trap “t-wave” CID cell with argon gas; the transfer collision cell was adjusted for 1 eV, using a scan time of 0.6 s and automatic interscan, both in low energy and high energy CID and an orthogonal acceleration time of light (oa-TOF). MSE was acquired over the m/z range of 50 - 1990. The RF offset (MS profile) was adjusted such that the LC/MSE data were effectively acquired from m/z 200 to 1990, which ensured that any fragment ions of m/z values less than m/z 200 observed in the LC/MSE data were known to arise from dissociations in the collision cell.

## MS data processing of proteomics

Raw data from 2D nano-UPLC/Q-TOF were analyzed by the ProteinLynx Global Server (PLGS 2.5) with ExpressionE informatics. A Swiss-Prot database (release 51.0, October 2013) was used for database searches and the search conditions were based on taxonomy of Homo sapiens, peptide tolerance, 15 ppm; fragment tolerance, 0.015 Da; trypsin missed cleavages, 1; minimum of consecutive fragment ions (y+, b+) for a peptide equal to 2; minimum of consecutive fragments for a protein equal to 5; minimum of peptides for a protein equal to 2; variable modifications, carbamidomethylation and Met oxidation, and the limitation of false-positive rate is 4%. Proteins identification were organized using PLGS into a list detailing protein unique to each condition and algorithm ratio between the different groups. Only proteins with attendance scores and confidence intervals higher than 99% according to the dedicated algorithm, respectively, were considered acceptable in these database searches.

## Quantitative analysis of proteomics

The analysis of quantitative changes in protein abundance, which is based on measuring peptide ion peak intensities observed in low collision energy mode in a triplicate set, was carried out using Waters Expression Analysis Software (WEPSTM), which is part of PLGS 2.2.5 (Expression version 2). For normalization, each sample was spiked with 125 fmol of tryptic digest of alcohol dehydrogenase 1 of yeast (ADH1, Waters) as an internal standard. Included were all protein hits that were identified with a confidence of 95%. Identical peptides from each triplicate set per sample were clustered based on mass precision 15 ppm, typically 5 ppm and a retention time tolerance of 0.25 min using clustering software included in PLGS 2.2.5. Protein scores were increased when the same peptide assignments were made in more than one replicate run. To avoid potential errors due to redundancies in assignments, searches were performed using the nonredundant Swiss-Prot database (as described above). Peptide probabilities are always softened slightly by the PLGS software prior to quantitation. Because of this, contributions from peptides with even 100% probability of presence were suppressed to avoid potential errors in quantitation. Normalization of the datasets was performed based on the spiked standard, but very similar results were obtained using the PLGS “auto normalization” function.

## Cell invasion assay

Cell invasion assay was performed as described ^2,3^. Invasion inserts with 8μm pore membranes from Corning (New York, USA) were coated with fibronectin from Sigma-Aldrich (Missouri, USA) as described ^4^. Cells were pretreated with vehicle or different concentration of 8u for 6 hours, respectively. The pretreated cells were seeded on the inserts to reach confluence in 12 hours, and then culture for another 24 hours with drug treatment. After fixed with 4 % formaldehyde, non-invading cells on the upper side of the membranes were removed by cotton swab. The invading cells were stained with 0.08 % trypan blue for 15 min as described^5^ and were photographed by a bright-field light microscope. Cell numbers of five random views were counted by Image-Pro-Plus 6.0 of Media Cybernetics (MD, USA).

## Western blotting

Cells were treated with vehicle or different concentration of 8u for 48 hours, whole cell proteins were extracted by RIPA lysis as described ^6^. Protein concentrations were measured by the BCA Protein Assay Kit from Beyotime (Shanghai, China). Equal amounts of protein were subjected to 12 % SDS polyacrylamide gel electrophoresis followed by transferring to PVDF membranes, and were subsequently analyzed with p18, p21, p27, CDK2, CDK4, CDK6, Cycline A, Cycline B1, Cycline D3, β-catenin, CLDN-1, E-cadherin, N-cadherin, Slug, Snail, Vimention, FASN, p-AKT and AKT antibodies from Cell Signaling (MA, USA) and HSP90α antibody from Gene Tex (CA, USA). Specific-appropriate secondary antibodies were detected and measured by the Luminescence Image Analyzer Tanon 5200 (Shanghai, China). The density of the bands were measured by Image Quant software (Molecular Dynamics, Sunnyvale, CA, USA).

## Immunofluorescence staining

Fixed treated cells with 4% paraformaldehyde for 25 min and permeabilized with 0.1% Triton X-100 for 10 min. Blocked with 2% bovine serum albumin (BSA) for 30 min at 37 °C and followed by the primary antibody against E-cadherin (1:200), β-catenin (1:100), Vimentin (1:100) and HSP90α (1:100) at 4 °C overnight. The cells were subsequently incubated with the corresponding Alexa 488-conjugated secondary antibody for 1 h at room temperature. The nuclei were stained with DAPI for 3 min. The images were captured using a DMI-4000B inverted fluorescence microscopy (Leica).

## Molecular docking

The molecular modeling of small molecule compound 8u was performed with the molecular modeling package SYBYL-X 1.3 (Tripos associate Inc., St. Louis, MO, USA) according to the reported process ^7^. Briefly, the three-dimensional coordinates of HSP90α protein were acquired from PDB (PDB ID: 3TUH). Only one of the two chains was kept and the protein chain was prepared for docking. The general procedure is as followed: (a) removing the water molecules which co-crystallized with the original protein structure; (b) preparing ligand and receptor and then ﬁnding the candidate binding site; (c) docking the test compound; (d) analysis of results.

## HSP90α siRNA transfection

Following 24 h of serum starvation, cells were transfected using siRNA-Mate^TM^ (GenePharma Co., Ltd.) reagent according to the manufacturer's protocol. Firstly, 11 µl (20 µM) HSP90α siRNA or negative siRNA as control that synthesized by Guangzhou GenePharma Co., Ltd. (Shanghai, China) were added into 100 µl serum-free DMEM, gently mix, placed at room temperature for 10min. Next, 16 µl siRNA-MateTM were diluted in the tube and mixed well immediately, gently centrifuged, placed at room temperature for 30 min. The complexes were added dropwise to each culture dish and incubated at 37 °C in an atmosphere containing 5% CO_2_ for 24 h. Following transfection, the cells were cultured in complete DMEM medium.

## HSP90α overexpression

HSP90α plasmid was constructed by genecreate (Wu Han, China). After ligation, the amplicon was transfected into puncture bacteria competent cells followed by plasmid extraction and transient transfection of HSP90α into HepG2 cells using polyplus as manual. After 12 h transfection, cells were replated and cultured for subsequent experiments.

# References

1 Perri, M. *et al.* Proliferative and anti-proliferative effects of retinoic acid at doses similar to endogenous levels in Leydig MLTC-1/R2C/TM-3 cells. *BBA-GEN. Subjects* **1800**, 993-1001 (2010).

2 Shan, D. D. *et al.* The G protein G alpha(13) is required for growth factor-induced cell migration. *Dev. Cell* **10**, 707-718 (2006).

3 Yang, S. Y. & Huang, X. Y. Ca2+ influx through L-type Ca2+ channels controls the trailing tail contraction in growth factor-induced fibroblast cell migration. *J. Biol. Chem.* **280**, 27130-27137 (2005).

4 Kleinman, H. K. Preparation of basement membrane components from EHS tumors (ed. Bonifacino, Juan S.) Unit 10. 2 (*Curr. Protoc. Cell Biol.,* 2001).

5 Clarke, G. D. Culture of Animal-Cells - a Manual of Basic Technique - Freshney,Ri. *Nature* **307**, 574 (1984).

6 Pfrepper, K. I. & Flügel, R. M. Molecular characterization of proteolytic processing of the Gag proteins of human spumaretrovirus. *J. Virol.* **73**, 7907-7911 (1999).

7 Zhang, C. *et al.* Exploration of (S)-3-aminopyrrolidine as a potentially interesting scaffold for discovery of novel Abl and PI3K dual inhibitors. *Eur. J. Med. Chem.* **46**, 1404-1414 (2011).

**Full‐length gels and blots**


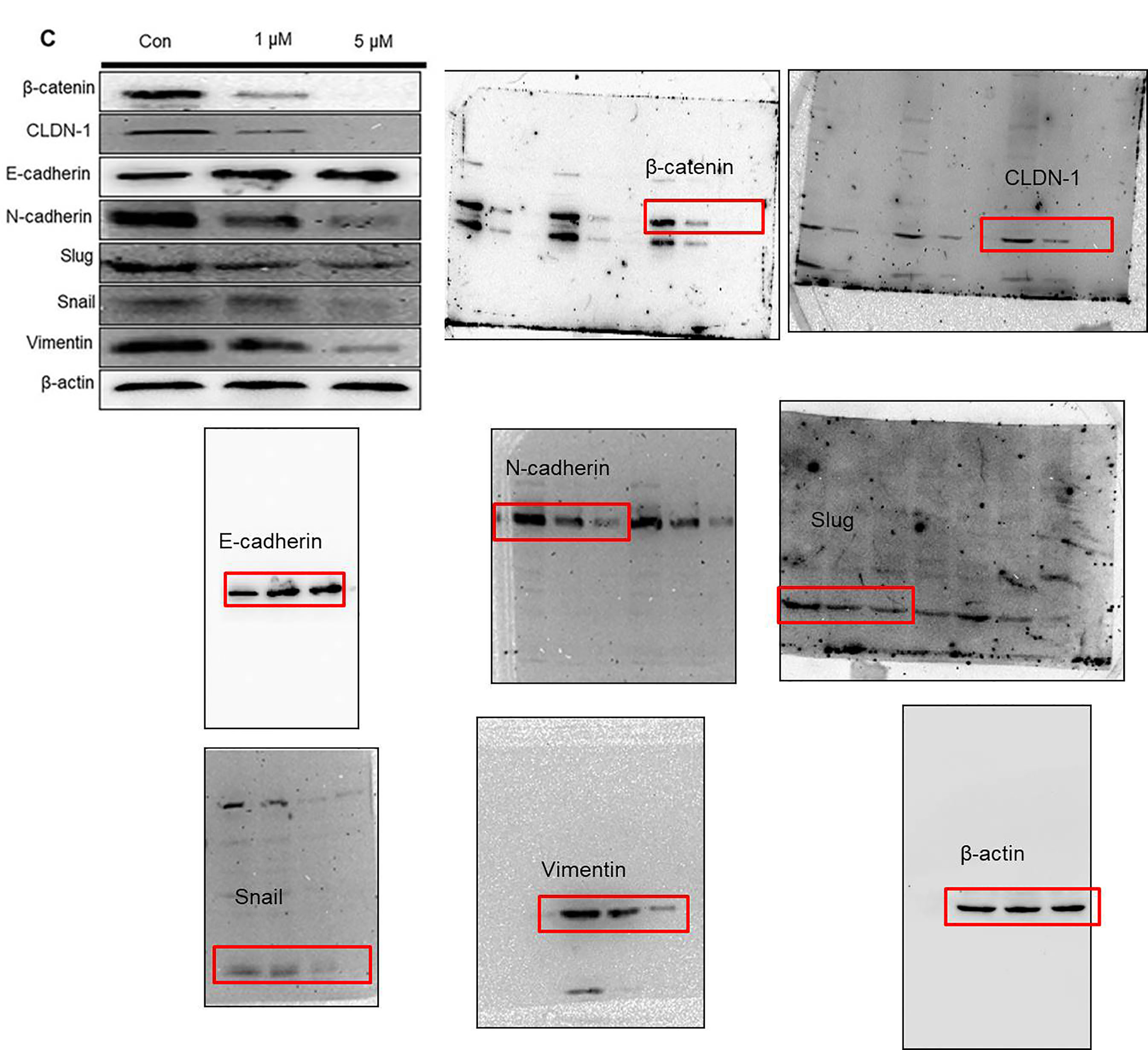


**Figure 2**


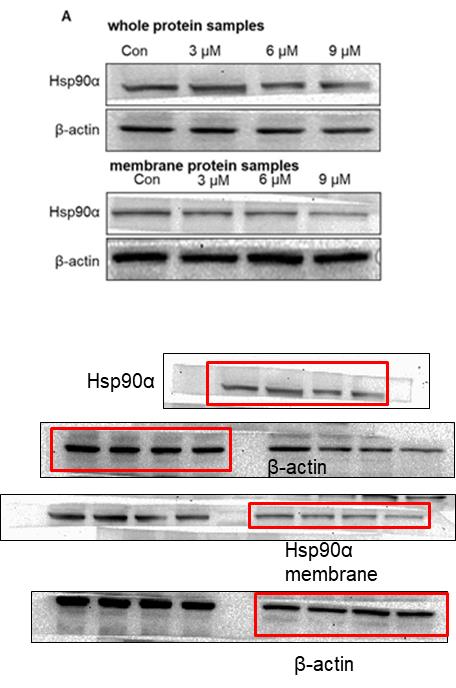


**Figure 4**


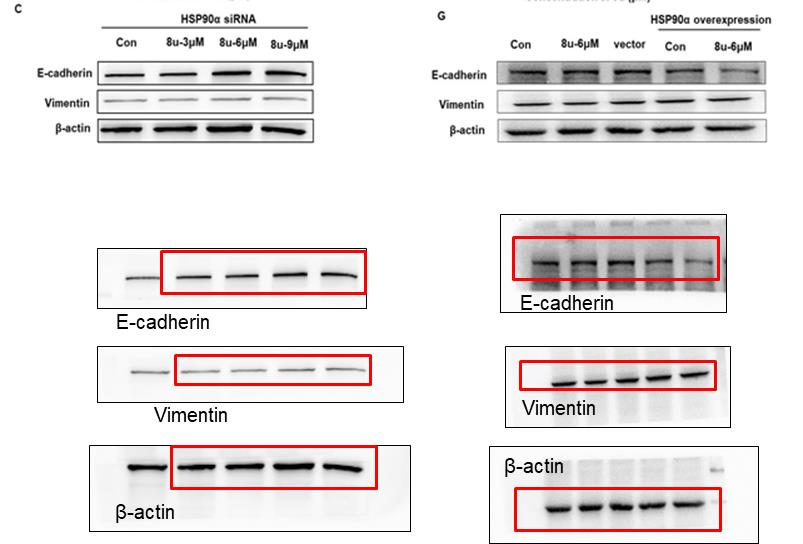


**Figure 6**


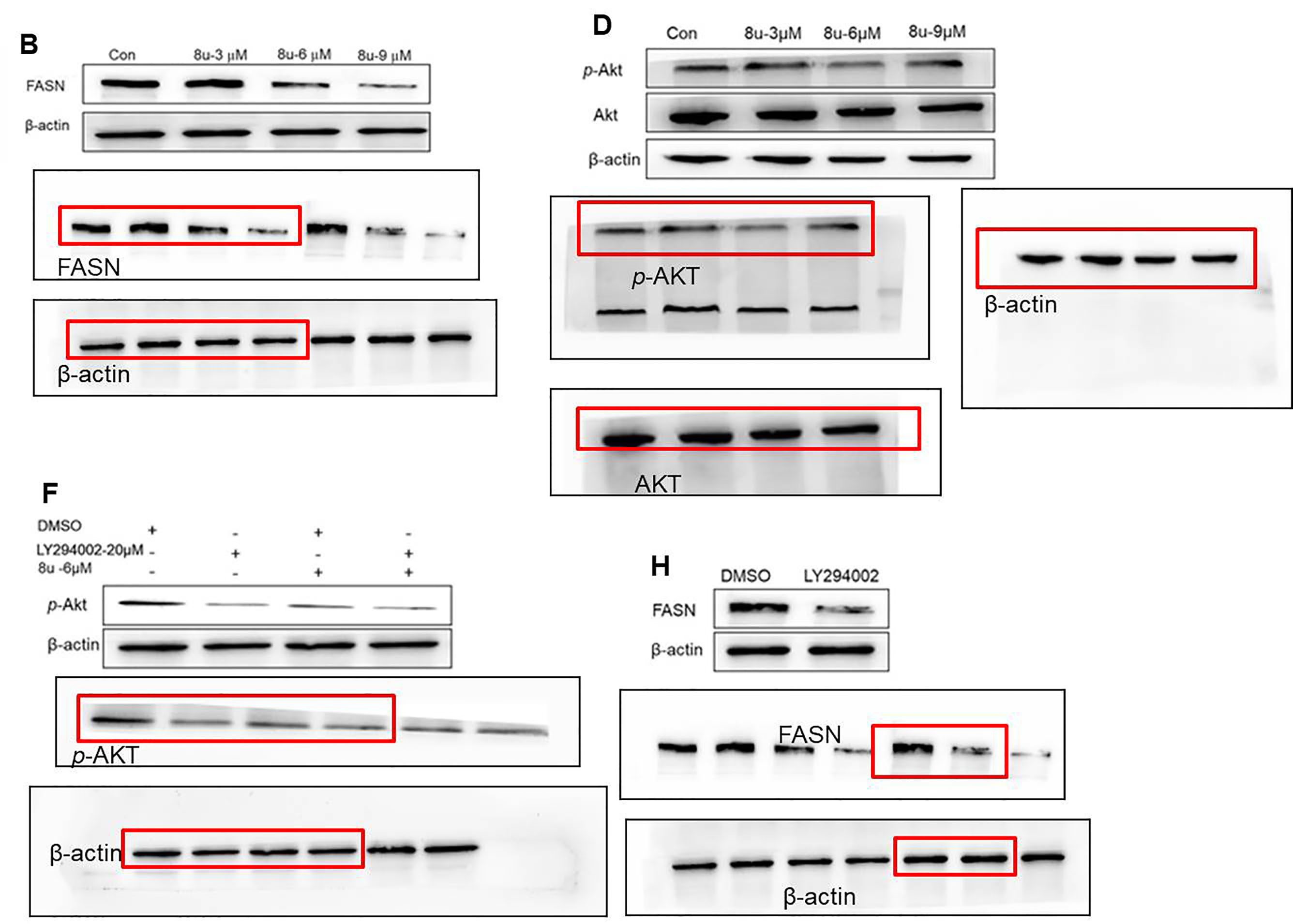


**Figure 7**

**
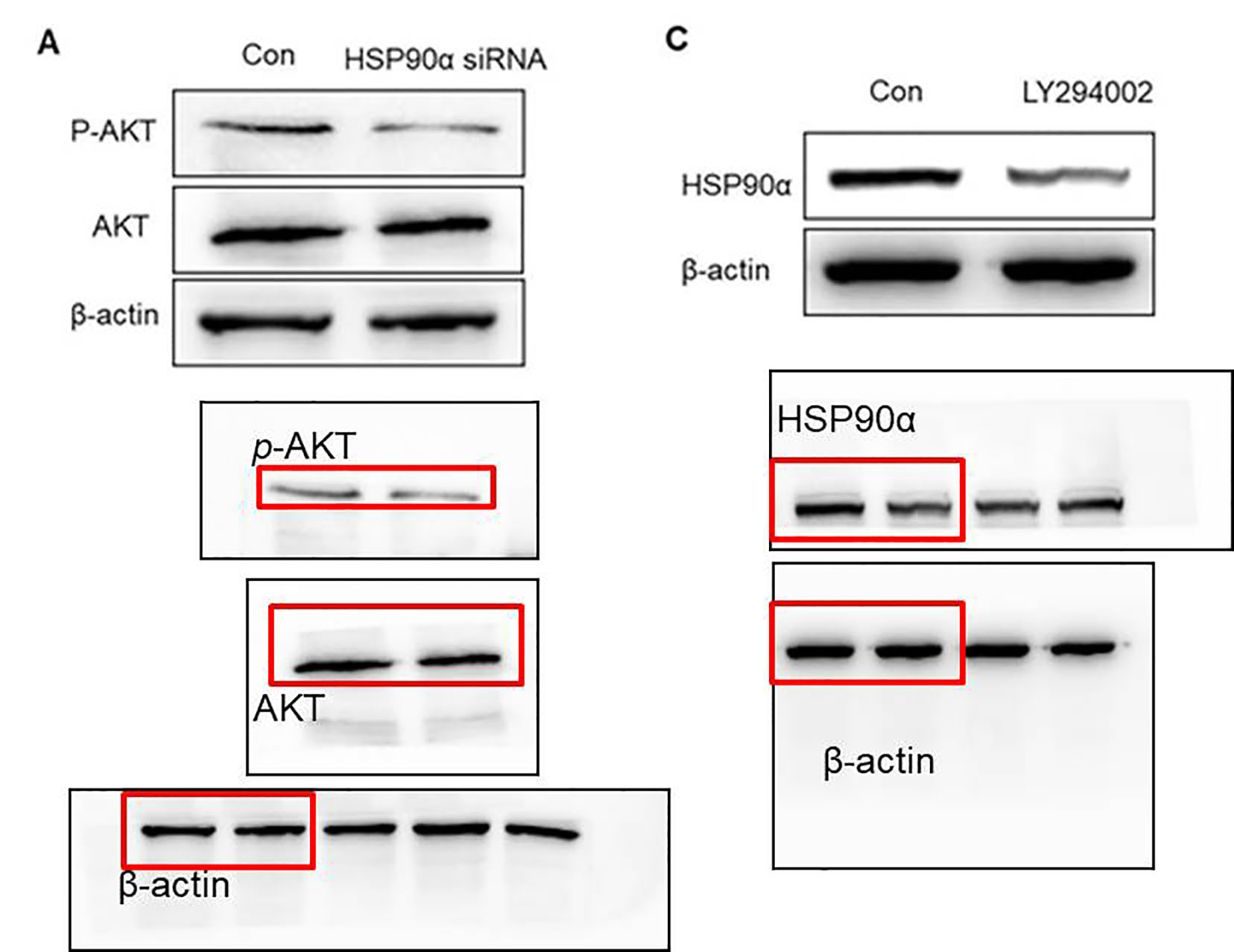
**

**Figure 8**


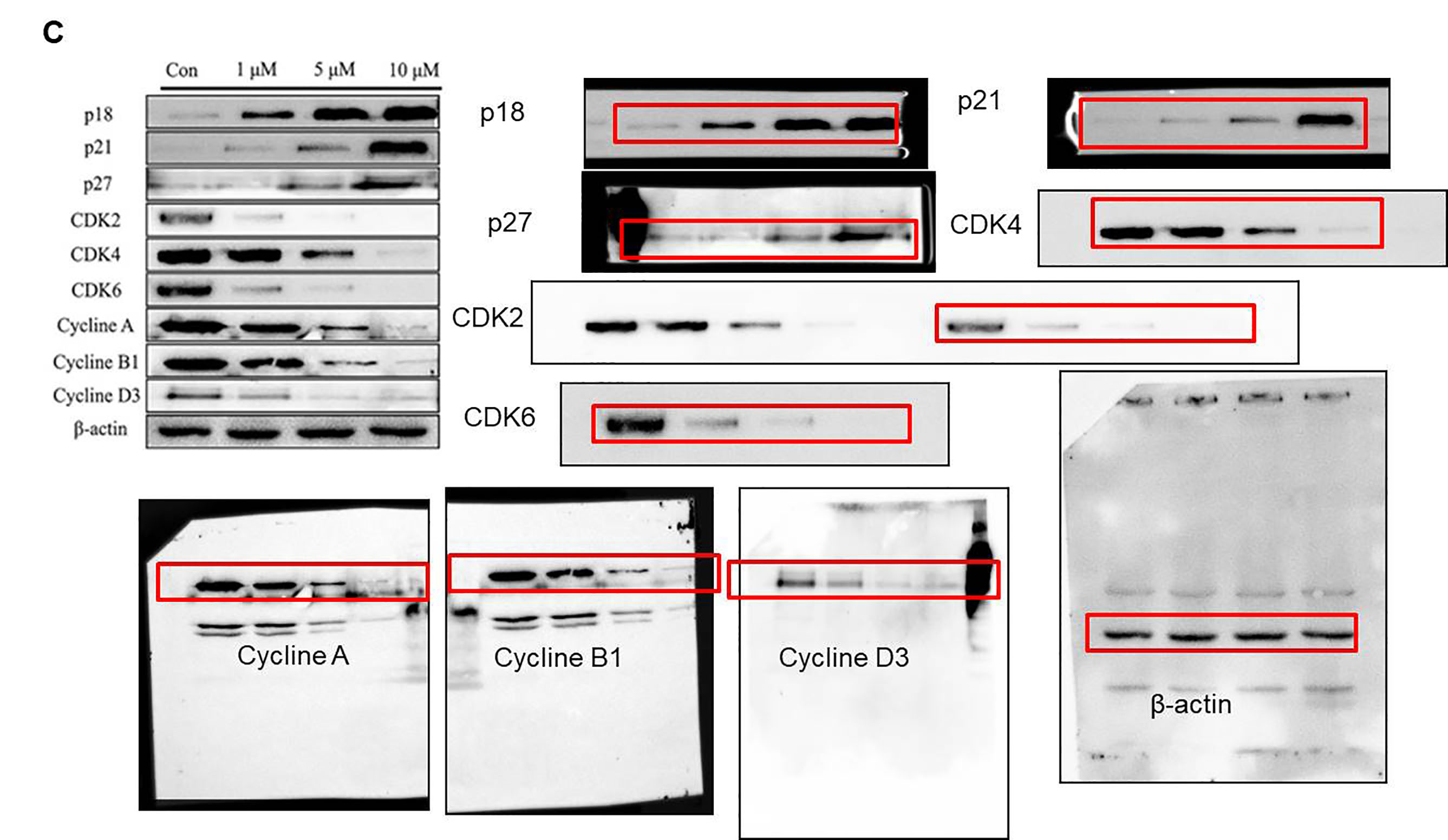


**Figure S2**


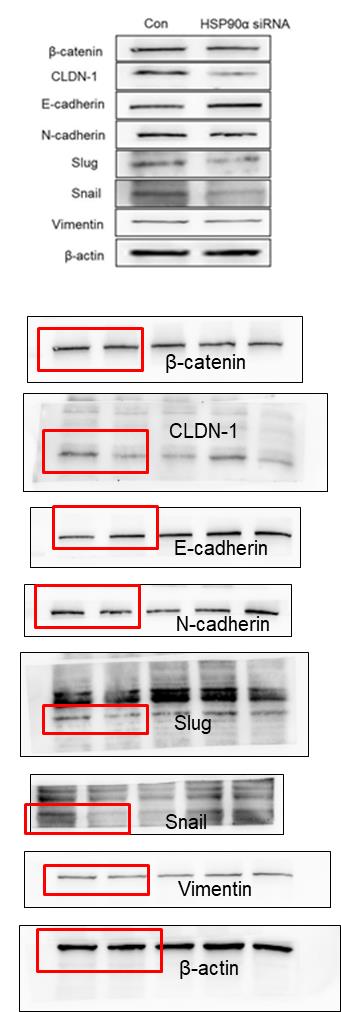


**Figure S5**


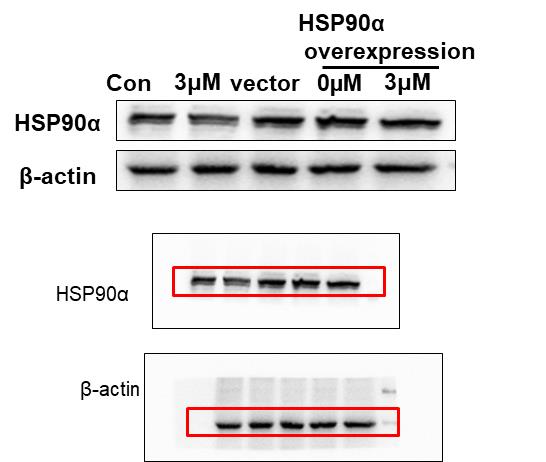


**Figure S6**


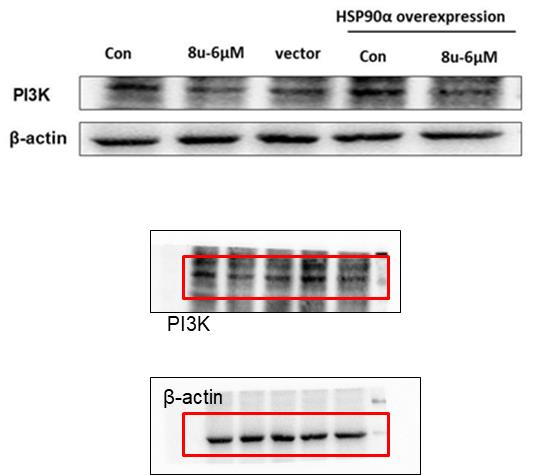


**Figure S7**
